# Human SIRT1 Multispecificity Is Modulated by Active-Site Vicinity Substitutions during Natural Evolution

**DOI:** 10.1093/molbev/msaa244

**Published:** 2020-09-21

**Authors:** Adi Hendler, Eyal Akiva, Mahakaran Sandhu, Dana Goldberg, Eyal Arbely, Colin J Jackson, Amir Aharoni

**Affiliations:** 1 Department of Life Sciences, Ben-Gurion University of the Negev, Be’er Sheva, Israel; 2 National Institute for Biotechnology in the Negev, Ben-Gurion University of the Negev, Be’er Sheva, Israel; 3 Department of Bioengineering and Therapeutic Sciences, University of California, San Francisco, CA; 4 Research School of Chemistry, Australian National University, Acton, ACT, Australia; 5 Department of Chemistry, Ben-Gurion University of the Negev, Be’er Sheva, Israel

**Keywords:** multispecificity, sirtuins, deacetylation

## Abstract

Many enzymes that catalyze protein post-translational modifications can specifically modify multiple target proteins. However, little is known regarding the molecular basis and evolution of multispecificity in these enzymes. Here, we used a combined bioinformatics and experimental approaches to investigate the evolution of multispecificity in the sirtuin-1 (SIRT1) deacetylase. Guided by bioinformatics analysis of SIRT1 orthologs and substrates, we identified and examined important amino acid substitutions that have occurred during the evolution of sirtuins in Metazoa and Fungi. We found that mutation of human SIRT1 at these positions, based on sirtuin orthologs from Fungi, could alter its substrate specificity. These substitutions lead to reduced activity toward K382 acetylated p53 protein, which is only present in Metazoa, without affecting the high activity toward the conserved histone substrates. Results from ancestral sequence reconstruction are consistent with a model in which ancestral sirtuin proteins exhibited multispecificity, suggesting that the multispecificity of some metazoan sirtuins, such as hSIRT1, could be a relatively ancient trait.

## Introduction

Protein–protein interaction (PPI) networks in multicellular organisms, such as humans, often include a larger number of cellular proteins relative to networks found in monocellular eukaryotic organisms (Evlampiev and Isambert 2008; Jancura et al. 2012; Jin et al. 2013; Liang et al. 2014). Conserved hub proteins, located at the heart of these PPI networks, must exhibit multispecificity to allow the recognition of the different partner proteins. Currently, little is known regarding the molecular basis and evolution of multispecificity in many PPI networks (Han et al. 2004). In some PPI networks, hub–partner interactions occur through a defined consensus recognition sequence found in different partners (Moldovan et al. 2007; Kim et al. 2009; Cino et al. 2013). However, in many PPI networks, shared recognition sequences/motifs are not observed and the molecular basis for hub–partner interactions is unknown. In these PPI networks, our understanding of the molecular basis and evolution of hub–partner interactions is very limited.

Many enzymes that catalyze the post-translational modification (PTM) of proteins are located at the heart of complex PPI networks (Beltrao et al. 2013). These enzymes catalyze the specific attachment or removal of different functional groups including phosphate, acetyl, methyl, as well as the protein ubiquitin. A substantial number of PTM-catalyzing enzymes, found in human cells, exhibit a remarkable ability to recognize many protein substrates and catalyze the modification of specific target residues. Such specificity allows the regulation of a variety of essential cellular processes including DNA replication, transcription and signal transduction (Deribe et al. 2010; Beltrao et al. 2013).

One of these enzymes is the human sirtuin SIRT1 (hSIRT1) that belongs to the large and ubiquitous family of sirtuin enzymes (Finkel et al. 2009). The sirtuins are NAD^+^-dependent deacetylases (Blander and Guarente 2004; Haigis and Guarente 2006). These enzymes are conserved from bacteria to humans and their overexpression in several eukaryotes was shown to increase organism’s life span (Howitz et al. 2003; Cohen et al. 2004; Kanfi et al. 2012). The yeast Sir2 (ySir2), from *Saccharomyces cerevisiae*, was the first member of the sirtuins to be characterized (Imai et al. 2000; Greiss and Gartner 2009). Subsequently, hSIRT1, which is a mammalian ortholog of ySir2, has become the most thoroughly characterized human sirtuin (Lavu et al. 2008). It is apparent that the number of hSIRT1 substrates is significantly higher than the number of substrates found for ySir2 (Chen et al. 2012; Martínez-Redondo and Vaquero 2013; Rauh et al. 2013; Bheda et al. 2016; Gil et al. 2017) with hundreds of substrates being characterized, including central regulatory proteins, such as p53, histones, FOXO, HSF1, and NFkB (Daitoku et al. 2004; Yeung et al. 2004; Westerheide et al. 2009). Despite extensive efforts, little is known of the molecular basis for how hSIRT1 recognizes target acetyl-lysine residues in such a wide variety of different substrates and no consensus sequence in hSIRT1 substrates has been identified (Blander et al. 2005; Garske and Denu 2006; Rauh et al. 2013). Thus, in addition to its important roles in human physiology and disease, hSIRT1 is an excellent model system for the study of the molecular basis and evolution of multispecificity in enzymes with a diverse set of substrates and cellular functions.

In this work, we have developed and applied a bioinformatics-experimental workflow to identify key residues in hSIRT1 that are important for its multispecificity. Our bioinformatics approach exploits natural variation in hSIRT1 homologs and ancestral sequence reconstruction (ASR) to identify variability in these key residues. Guided by the bioinformatics analysis, we generated different hSIRT1 mutants at residues in the immediate vicinity of the active site. Examination of these mutants revealed that, for many of these mutants, the deacetylation activity with conserved histones was maintained, but the activity with p53 K382Ac, which is present in Metazoa but not Fungi, was greatly reduced. Our study reveals that several residues surrounding the active site are essential for hSIRT1 multispecificity. Our results are consistent with a model in which multispecificity was present in ancestral sirtuins and maintained in metazoans but was lost during the evolution of fungal sirtuins. Thus, these results indicate that fungal sirtuins have become more specialized, relative to the common ancestor.

## Results

### A Bioinformatics Workflow for Identifying Candidate Amino Acid Positions Important for SIRT1 Multispecificity

To identify a limited number of candidate positions that may be important for hSIRT1 multispecificity, we combined sequence similarity networks (SSN) (Atkinson et al. 2009; Akiva et al. 2017), multiple sequence alignments (MSA), and phylogenetic trees focusing on the deacetylase (DAC) domain of SIRT1. The construction of reliable MSAs of SIRT1 homologs requires homogenous sampling of SIRT1 sequence space. Thus, we first delineated the sequence-similarity boundary that differentiates SIRT1 family members from the larger sirtuin superfamily. We constructed an SSN of the entire sirtuin superfamily including 9,521 sequences and then mapped specific sirtuins, documented in the literature, to identify the different sirtuin classes (SIRT1–7). This representation enabled the delineation of SIRT1 subgroup containing 1,107 sequences that can be clearly separated from other sirtuin groups for full-length sirtuins (supplementary fig. S1, Supplementary Material online) and for the DAC domain (supplementary fig. S2, Supplementary Material online). To generate a SIRT1 MSA, we used the Enzyme Function Initiative Enzyme Similarity Tool (EFI-EST) to collect sequences related to hSIRT1 (Gerlt et al. 2015). Using incremental percent identity edge cutoffs in Cytoscape (Shannon et al. 2003), clusters delineated by sirtuin subfamily were obtained. The final set of 151 SIRT1 sequences was aligned using PROMALS3D (Pei et al. 2008) to generate the MSA (see details in Materials and Methods).

The MSA was used to generate a phylogeny of these proteins, which was compared with the established species-level phylogeny for the taxa from which we obtained protein sequences. This revealed that the unconstrained tree topology (supplementary fig. S3, Supplementary Material online), although fully consistent with the corresponding species tree in terms of the split of fungi and metazoa, as well as the topology of the fungi clades, was not concordant with the species-level tree within the Metazoan clades (Philippe et al. 2005; dos Reis et al. 2015) (supplementary fig. S4, Supplementary Material online). In the absence of evidence for hemiplasy (Maddison 1997; Avise and Robinson 2008) or horizontal gene transfer (Maddison 1997), this discordance is most likely an artefact of the sequence data (an in-depth discussion of possible causes for this discordance is provided in the legend of supplementary fig. S4, Supplementary Material online). Accordingly, we constrained the tree search space to enforce a species-concordant topology to ensure that any analysis or reconstructions of ancestral sequences would be better approximations of the ancestral proteins. A phylogeny with a species-concordant topology (supplementary fig. S5, Supplementary Material online) was produced by supplying a constraint tree (supplementary fig. S4, Supplementary Material online) to the inference algorithm in IQ-TREE. Using statistical tests, we validated that the constrained topology was not significantly different from the original unconstrained maximum likelihood (ML) phylogeny (supplementary table S1, Supplementary Material online). Thus, by constraining the tree search space, we have generated a superior phylogeny that is both concordant with the well-established species-level tree and is equally well-supported by the MSA data as the discordant unconstrained ML phylogeny.

**Fig. 1.**
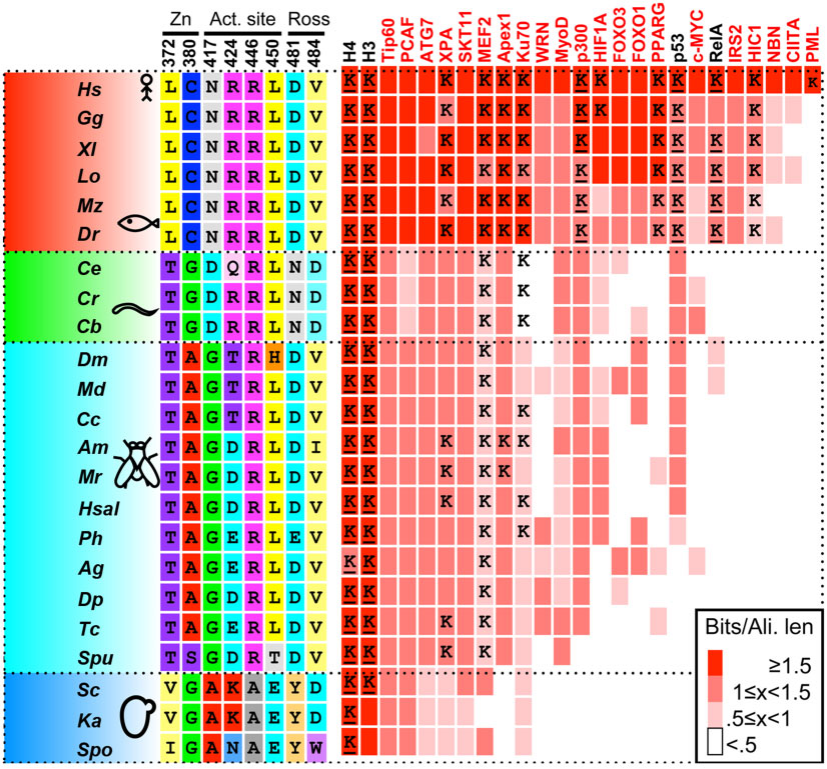
SIRT1 evolution is correlated with substrate repertoire expansion. SIRT1 orthologs are shown on the left, representing major branches of eukaryotic taxa: vertebrates (red), invertebrates I (green, mainly mullusca, and nematodes), invertebrates II (cyan, mainly Ecdysozoa [insects]), and fungi (blue). The eight positions in hSIRT1 selected for substitutions (see text and figs. 2 and 3) demonstrate conservation that is associated with the major evolutionary transitions. Right (red), a matrix depicting the conservation of 25 hSIRT1 substrates in eukaryotes. The substrates tested in this study are shown in black headings. The conservation level of the orthologs, measured by Bit score divided by alignment length is color-coded from white (low conservation) to red (high conservation). The appearance of orthologs in the different organisms is cross-validated by the inParanoid (Sonnhammer and Östlund 2015) and EggNOG (Huerta-Cepas et al. 2016) databases, as well as literature review and manual examination. In cases where the acetyl lysine position is known for the hSIRT1 human substrate, a “K” appears in the relevant matrix cell. Underlined “K” represents the substrate column tested in this study.

**Fig. 2.**
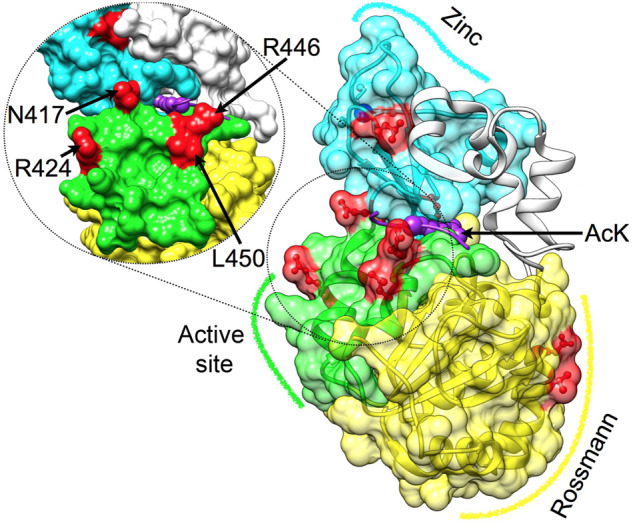
Mapping semiconserved positions on the DAC of hSIRT1. Three major structural regions in hSIRT1 are shown (PDB 4KXQ) (Davenport et al. 2014), along with the specific positions that were substituted (red), Rossmann-like domain (yellow, positions 481 and 484), the active-site region (green, positions 417, 424, 446, and 450, also tilted and enlarged on the left) and the zinc-binding domain (cyan, positions 372 and 380, the zinc ion is in blue). The peptide with the acetyl lysine is shown in purple, its approximate position is based on Sir2-p53 complex (PDB 2H4F) (Hoff et al. 2006). This position highlights the proximity between positions located at the active-site region and the peptide.

To identify semiconserved residues in SIRT1 that may be important for its multispecificity, we used structural information, SIRT1 tree and MSA. We filtered all DAC positions, containing 268 residues, based on surface accessibility, Shannon entropy analysis (Stewart et al. 1997) and physicochemical amino acid properties and remained with 37 possible positions (see detailed description in supplementary materials and methods, Supplementary Material online). These positions were further reduced to 8 based on visual inspection of hSIRT1 structure, (Davenport et al. 2014) identifying proximal pairs or quadruple positions in the Rossmann-like domain (positions 481 and 484), the active-site region (positions 417, 424, 446, and 450) and the zinc-binding domain (positions 372 and 380, figs. 1 and 2).

### The Evolutionary Dynamics of SIRT1 Substrate Expansion

Characterization of the evolutionary dynamics of SIRT1 substrate expansion can be used to identify correlations between amino acid substitutions in SIRT1 and substrate expansion (fig. 1). To this end, we first collected 25 experimentally validated substrates of hSIRT1 for which their acetylated lysine is at least partially known (Chen et al. 2012; Rauh et al. 2013) (supplementary table S2, Supplementary Material online). Next, we performed phylogenetic profiling for these substrates, that is, assessing the presence or absence of orthologs within species used to construct the SIRT1 MSA and phylogenetic tree (see Materials and Methods for details). In addition, we generated MSAs of these orthologs, to evaluate the conservation level of the target acetyl-lysine in these SIRT1 substrates (for example, see supplementary fig. S6, Supplementary Material online). As expected, we found that histones are completely conserved SIRT1 substrates in all organisms, whereas acetylated p53 and RelA are examples of substrates that appeared in complex eukaryotes (fig. 1). In almost all cases, we identified the appearance of the substrate, followed by fixation of a lysine residue that was shown to be acetylated in the human substrate (fig. 1 and supplementary table S2, Supplementary Material online). This classification allowed us to select specific substrates for our experimental analysis (see below) and to identify correlations between the substitutions identified in hSIRT1 and the expansion of the substrate repertoire (fig. 1).

### Experimental Analysis of hSIRT1 Mutants Indicates Functional Compensation between Mutations

**Fig. 3.**
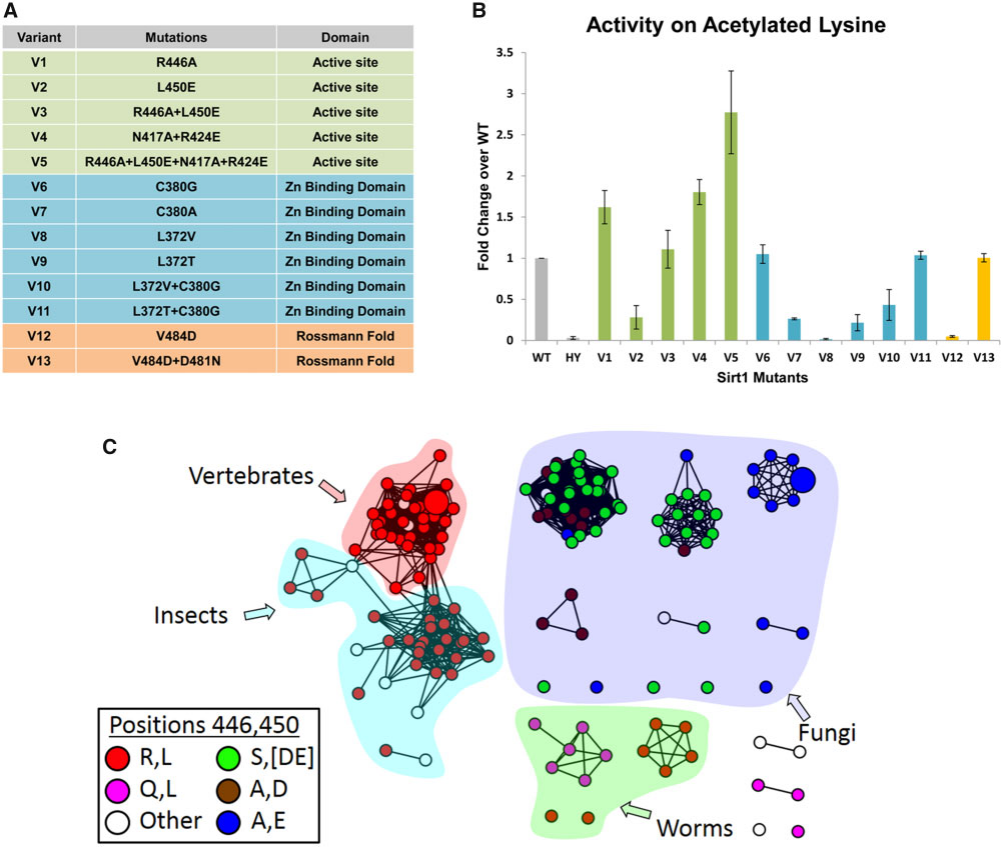
The analysis of 13 hSIRT1 variants revealing functional compensation between mutations in hSIRT1. (*A*) A list of the 13 hSIRT1 mutants generated (V1–V13) classified according to three structural regions in hSIRT1: The active-site vicinity (green), zinc-binding domain (blue) and Rossmann-like domain (orange) (see also fig. 2). (*B*) Activity analysis of V1–V13 using the FDL assay using fluorogenic *N*-acetyl lysine. The slopes derived from the FDL experiments were normalized relative to the WT hSIRT1 and results are presented as fold change over the WT (for representative kinetic curves see supplementary fig. S7, Supplementary Material online), HY is the inactive hSIRT1 mutant containing the H363Y mutation. Values shown are the mean of three independent repeats whereas the error bars represent the standard deviation from the mean (*C*). An SSN of 151 SIRT1 sequences showing the correlated substitutions between positions 446 and 450 in hSIRT1 orthologs. The coloring of each node (sequence) is by the identity of positions 446 and 450. The first letter is position 446 and the second is 450, for example, red node color represents a sequence containing R at position 446 and L at position 450. The majority of SIRT1 sequences from vertebrates, insects, and worm species contain R at position 446 and L at position 450 (red) whereas most fungi species contain A at position 446 and E at position 450 (blue). The proximity between these positions in hSIRT1 is shown in figure 2.

Based on the bioinformatics and structural analysis described above, we generated, expressed and purified 13 hSIRT1 mutants (V1–V13) containing 1–4 substitutions in different regions of the DAC domain (figs. 2 and 3). The deacetylation activity of the purified mutants was measured with fluorogenic *N*-acetyl-lysine using the Fluor-de-Lys (FDL) assay (Wegener et al. 2003) and compared with the activity of WT hSIRT1. This is the most simple substrate for SIRT1 orthologs; consisting of an acetylated lysine conjugated to a fluorescent group. Thus, it is useful to detect whether the mutant enzymes are catalytically active, or not, but cannot be used to make inferences regarding substrate specificity. The FDL analysis allowed us to examine the activity of each variant with the *N*-acetyl-lysine relative to the WT and reveal functional compensation between mutations. We found that the L450E mutation (V2) shows low deacetylation activity relative to the WT. However, the double L450E R446A mutant (V3) exhibits deacetylation activity that is similar to WT; indicating functional compensation between these two mutations (fig. 3). Interestingly, the quadruple mutant containing N417A/R424E and R446A/L450E (V5) exhibits a significantly increased deacetylase activity of ∼3-fold relative to the WT (fig. 3 and supplementary fig. S7, Supplementary Material online). Thus, V5, which contains four active-site vicinity mutations that are mainly observed in Fungi SIRT1 orthologs (fig. 1), exhibits significantly enhanced activity toward *N*-acetyl-lysine.

We found additional functional compensation between mutations in the zinc-binding domain and the Rossmann-like domain (fig. 2). In the zinc-binding domain, we found that the single L372T mutants (V9) are inactive. However, the L372T/C380G double mutant (V11) exhibits deacetylase activity that is similar to the WT (fig. 3). Similarly, although the single V484D mutant (V12) is inactive, the V484D/D481N double mutant (V13) in the Rossmann-like domain exhibits deacetylase activity that is similar to the WT (fig. 3). Structural analysis of positions D481 and V484 suggests that the negative charge introduced by the V484D mutation is probably compensated for by the loss of the negative charge due to the D481N mutation (supplementary fig. S8, Supplementary Material online).

Next, we examined co-evolutionary coupling between these residues using Generative REgularised ModeLs of ProteiNs (GREMLIN) analysis of DAC domain sequences (Balakrishnan et al. 2011). We found strong co-evolutionary coupling between R446/L450, located in the vicinity of the hSIRT1 active site, and D481/V484, located in the Rossmann-like domain, suggesting that co-evolution between these residue pairs is functionally important for hSIRT1 deacetylase activity (fig. 3). The GREMLIN scaled score for 446/450 and 481/484 pairs was 2.28 and 2.22, respectively, that is much above the 1 threshold for the average score (Balakrishnan et al. 2011). However, this analysis did not detect co-evolution between L372/C380 within the zinc-binding domain and N417/R424 in the vicinity of SIRT1 active site.

### hSIRT1 Variants Containing Active-Site Substitutions Maintain Activity toward Conserved Histone Substrates

**Fig. 4.**
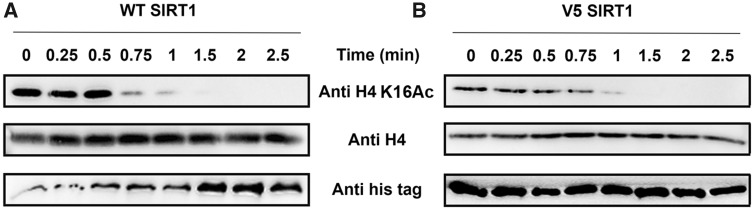
The activity of V5 toward acetylated histones is maintained. Western blot kinetic analysis of WT (*A*) and V5 (*B*) activity toward H4K16Ac in the context of native histones shows that V5 is fully active toward this substrate. SIRT1 deacetylation activity is detected by the time dependent decrease in H4K16Ac signal. H4K16Ac, H4, and hSIRT1 levels were detected using anti-H4K16Ac antibody, anti-H4 antibody, and anti-6xHis antibody, respectively.

As described above, the hSIRT1 V3/V4/V5 mutants, in which amino acids have been substituted by residues found in Fungi or fly SIRT1 orthologs, exhibit activity that is comparable to (or higher) than WT hSIRT1 with the *N*-acetyl-lysine substrate (fig. 3). To examine V3/V4/V5 activity with an acetyl lysine in universally conserved histone proteins, we first purified the chromatin fraction from HEK293T mammalian cells. Next, to examine the kinetics of histone deacetylation, we incubated V3/V4/V5 with the purified chromatin fraction for specific time durations. We then utilized western blot analysis to monitor hSIRT1 activity by following the time dependent decrease in H4K16Ac signal. We found that V3/V4/V5 activity toward H4K16Ac in the chromatin fraction is similar to WT hSIRT1 (fig. 4 and supplementary fig. S9, Supplementary Material online). These results show that V3/V4/V5 maintain full activity with the conserved H4K16Ac. We further validated these results by examining the activity of the different hSIRT1 variants toward peptides containing H3K9Ac, H3K56Ac and H4K16Ac derived from human histones H3 or H4. SIRT1 activity with these peptides was measured using an ammonia-coupled assay as previously described (Smith et al. 2009; Meledin et al. 2013). This continuous spectroscopic assay allows determination of the Michaelis Menten kinetic parameters for the reaction. Analysis of these variants with the different peptides (table 1 and supplementary table S3, Supplementary Material online) revealed similar or in some cases even lower *K*_M_ values for the V3/V4/V5 variants, relative to the WT (table 1, supplementary table S3 and figs. S10 and S11, Supplementary Material online). Overall, this analysis indicates that the mutations in V3/V4/V5 do not compromise hSIRT1 ability to recognize and catalyze the deacetylation of the conserved acetylated histones.

**Table 1.** Kinetic Parameters of WT and V5 with Different Acetylated Peptides.^a^

SIRT1	WT			V5		
Parameters Peptide	*k*_cat_ (s^-1^)	*K*_M_ (µM)	*k*_cat_/*K*_M_ (mM^-1^ s-^1^)	*k*_cat_ (s^-1^)	*K*_M_ (µM)	*k*_cat_/*K*_M_ (mM^-1^ s^-1^)
H4K16	0.2 ± 0.04	52.6 ± 18.5	5 ± 2.3	0.2 ± 0.01	8.8 ± 3.7	26.8 ± 5.2
H3K9	0.3 ± 0.07	101.6 ± 31.4	3.7 ± 2.2	0.1 ± 0.03	47 ± 24.5	2.6 ± 0
H3K56	0.1 ± 0.01	20.5 ± 7.6	7.5 ± 2.3	0.1 ± 0.01	19.4 ± 8.3	6.5 ± 1.9
p53	0.4 ± 0.02	17.4 ± 3.8	25.8 ± 7.1	0.3 ± 0.02	42.2 ± 6.2	8.2 ± 3.5
p65	0.1 ± 0.01	3.6 ± 2.6	44.5 ± 4.6	0.3 ± 0.04	30.1 ± 12.8	9.9 ± 3.7

a Parameters were derived from fitting the kinetic measurements of the different hSIRT1 variants to the MM equation. For complete parameters of V3 and V4 see supplementary table S3, Supplementary Material online and for peptide sequences see supplementary table S4, Supplementary Material online.

### Multiple Active-Site Substitutions Lead to Reduced Activity toward Acetylated p53

Our bioinformatics analysis of SIRT1 substrates indicates that although histones are fully conserved, p53 is less conserved and the fixation of K382 appears only in multicellular organisms (Joerger et al. 2014) (fig. 1). We hypothesized that residues N417, R424, R446, or L450 (figs. 1 and 2) may be important for efficient recognition of acetylated p53 protein by hSIRT1. Accordingly, the V3/V4/V5 mutants, containing two or four active-site vicinity mutations observed mainly in the Fungi SIRT1 orthologs (figs. 1–3), may exhibit decreased deacetylation activity toward p53.

**Fig. 5.**
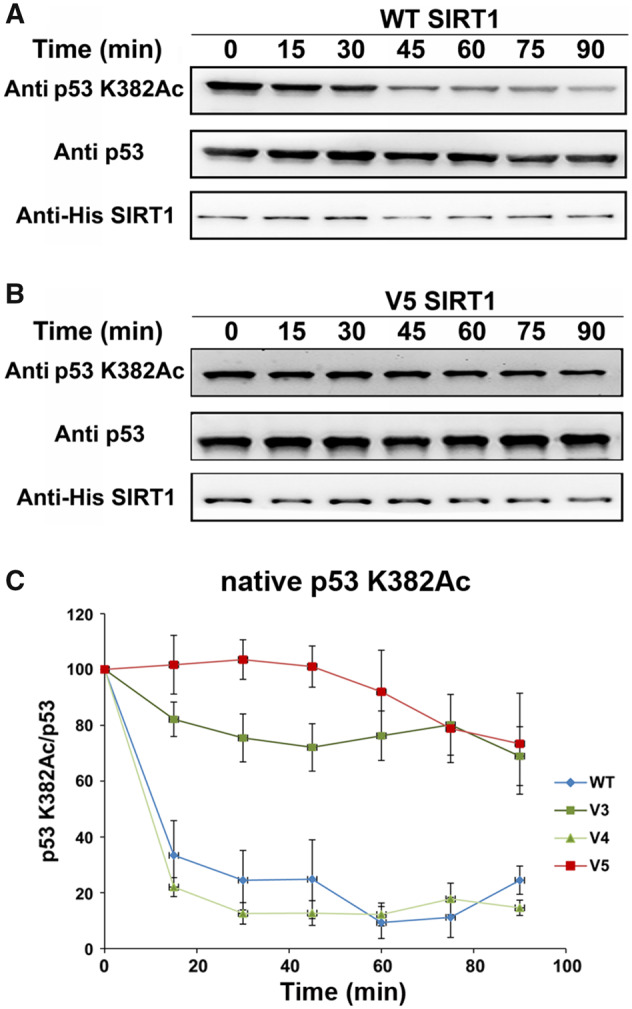
The activity of WT and V3–V5 with acetylated p53. Western blot analysis of WT (*A*) and V5 (*B*) activity toward p53-K382Ac in the context of native p53 containing AcK at position 382. Analysis of p53-K382Ac, p53, and hSIRT1 levels was performed using anti-K382Ac antibody, anti-p53 antibody, and anti-6xHis antibody, respectively. (*C*) Quantification of the western blot kinetic analysis of WT and V3–V5 variants shows that V3 and V5 exhibit significantly reduced activity, relative to the WT. The time dependent decrease in K382Ac signal was normalized to time 0 and shown as the percentage of p53-K382Ac/p53 band signals. Each experiment was performed in triplicates. Representative blots for V3 and V4 activity are shown in figure S12. Band intensities were quantified by Image J.

To examine this hypothesis, we measured V3/V4/V5 activity toward acetylated p53 protein. We first expressed and purified recombinant human p53 protein in *Escherichia coli*, in which we used genetic code expansion (unnatural amino acid mutagenesis) to site specifically incorporate *N*-(ε)-acetyl-l-lysine (AcK) into position K382 during protein translation (Neumann et al. 2009; Avrahami et al. 2018). We then used western blot analysis to monitor the time dependent deacetylation activity of the hSIRT1 variants with the K382Ac p53 protein. Using this assay, we observed a significant decrease in V3 and V5 activity toward acetylated p53 protein, relative to the WT, whereas the activity of V4 was maintained (fig. 5 and supplementary fig. S12, Supplementary Material online). This analysis revealed that the R446A and L450E mutations in V3 lead to a significant decrease in p53 activity whereas the N417A and R424E mutations in V4 do not affect this activity. These results show that although V3 and V5 maintain full activity toward histones (fig. 4), their activity toward p53 protein substrate is significantly reduced (fig. 5), presumably due to the effect of the R446A and L450E mutations.

Next, we examined the deacetylation activity of V3/V4/V5 with a K382Ac peptide derived from human p53. We found that, in contrast to the assays with full length protein, all proteins retained activity (*k*_cat_/*K*_M_ within ∼3-fold of WT) with the peptide, although the *K*_M_ of V5 toward this peptide is increased by ∼2.5-fold, relative to the WT, indicating some decrease in affinity of V5 to the p53 peptide (table 1 and supplementary fig. S11, Supplementary Material online). Finally, to examine whether the V3/V4/V5 variants exhibit altered activity with an additional substrate that appeared later in evolution, we examined their activity with an acetylated peptide derived from human RelA/p65 K310Ac (Yeung et al. 2004) (fig. 1). We found that V5 exhibited up to 10-fold higher *K*_M_ values with the RelA/p65 K310Ac peptide than WT hSIRT1 indicating lower affinity toward this peptide (table 1 and supplementary fig. S11, Supplementary Material online).

### Ancestral Sequence Reconstruction (ASR) of hSIRT1

**Fig. 6.**
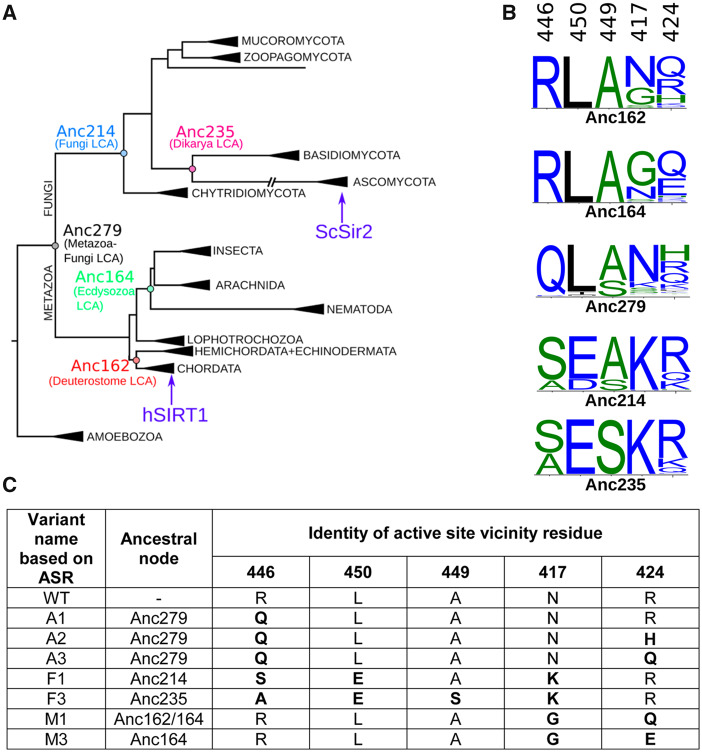
Ancestral sequence reconstruction (ASR) of SIRT1. (*A*) The constrained maximum-likelihood phylogeny of the SIRT1 DAC domain inferred from an alignment of 142 sequences. Key ancestral nodes are indicated. Subtrees are collapsed and labeled according to taxonomic kingdom (e.g., Amoebozoa), phylum (e.g., Ascomycota), or class (e.g., Insecta). Human SIRT1 is found in the Chordata subtree; ySIR2 is found in the Ascomycota subtree. (*B*) The posterior probability distribution at the putative active-site vicinity residues (446, 450, 449, 417, and 424) shown as sequence logos for key ancestral nodes. Sequence logos constructed using WebLogo 3 (Crooks et al. 2004). (*C*) Variants of hSIRT1 containing possible combinatorial mutations based on ASR analysis. These variants were generated in the lab and experimentally tested (see text and fig. 7).

The loss of activity observed in the V3 and V5 variants toward p53 suggests two possibilities regarding the evolutionary dynamics of SIRT1 activity toward this substrate: 1) ancestral SIRT1 proteins may exhibit activity toward p53 indicating that this activity was maintained in Metazoan but was lost in Fungi sirtuins, that is, the fungi sirtuins have evolved higher specificity; or 2) ancestral SIRT1 proteins may exhibit no activity toward p53 and the multispecificity of hSIRT1 is an evolved trait that was gained during the evolution of Metazoan sirtuins. To examine these possibilities, we performed ASR analysis of the SIRT1 DAC domain, focusing on positions within the vicinity of the active site: specifically 417, 424, 446, and 450 (already examined in V5), as well as position 449 (fig. 6). We used ASR to reconstruct SIRT1 ancestral sequences to the extant Fungal and Metazoan orthologs, with a particular focus on the five different positions listed above (see details in Materials and Methods and fig. 6).

### Experimental Analysis of ASR-Based hSIRT1 Mutants

Based on the ASR analysis, we generated, expressed and purified additional set of hSIRT1 variants containing active-site substitutions at positions 446, 450, 417, 424, and 449 according to their putative identities in the ancestral node sequences (fig. 6). These variants incorporate ambiguity in the reconstructions, that is, in some positions, the probability of a particular amino acid is <1, in which case the two most probable amino acid substitutions were tested. These variants include substitutions from representative ancestors on the Metazoan branch (M1 and M3; representing Anc164), the Fungi branch (F1 and F3; representing Anc235 and Anc214), and the Metazoa–Fungi common ancestor (A1–3; representing Anc279) (fig. 6). Previous studies indicated that a shorter version of hSIRT1 containing residues 236–684 (Knyphausen et al. 2016) leads to reduced aggregation with complete maintenance of SIRT1 enzymatic activity. To examine the shorter version of hSIRT1 in our system, we analyzed the activity of truncated WT and V5 with acetylated p53 protein. We found that these variants exhibit similar activity profiles with p53 as the full-length respective proteins (supplementary fig. S13, Supplementary Material online). Thus, we constructed the ASR variants in the background of the truncated hSIRT1 variant for functional analysis (fig. 6).

**Fig. 7.**
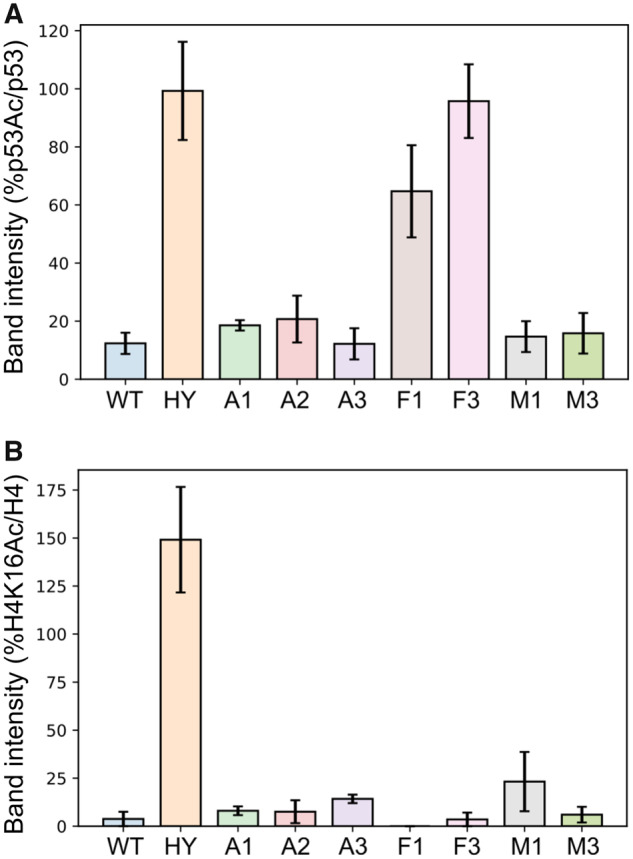
Quantification of western-blot activity assay for the different hSIRT1 ASR based variants with native p53 K382Ac and native H4K16Ac. In this graph, because the acetylated substrate was quantified, high band intensity corresponds to low substrate turnover, that is, low activity. (*A*) Data for p53 K382Ac were quantified by the band intensity following membrane exposure to anti-p53Ac antibody at 120 min time point as a percentage of the intensity of the same time point probed with anti-p53, normalized to the percentage of p53 K382Ac/p53 band intensities at *t* = 0. (*B*) Data for H4K16Ac are quantified by the band intensity following membrane exposure to anti-H4K16Ac antibody at 180 s time point as a percentage of the intensity of the same time point probed with anti-H4, normalized to the percentage of H4K16Ac/H4 band intensities at *t* = 0. The hSIRT1 HY mutant, containing the catalytic H363Y mutations, served as the negative control. Error bars show the standard error from the mean (SEM) for a minimum of 2 experimental repeats. Representative western blots for all variants are shown in supplementary figure S15, Supplementary Material online.

To examine the activity of the different ASR variants, we first used the FDL assay, as described above, to verify that all variants are active (supplementary fig. S14, Supplementary Material online). Next, we used western blot analysis to examine the deacetylation activity of the different variants with H4K16Ac in native histones and K382Ac in p53 protein. Interestingly, we found that the A1–A3 variants, corresponding to the possible residue configuration of the protein in the Metazoa–Fungi common ancestor (Anc279), exhibited similar activity with both of these substrates as WT hSIRT1 (figs. 6 and 7 and supplementary fig. S15, Supplementary Material online). In contrast, we found that the F1 and F3 variants, corresponding to possible residue configurations of the common ancestor of all Fungi-Dikarya (Anc235/214), maintained activity with the conserved H4K16Ac but exhibit significant loss of activity with the p53 K382Ac (figs. 6 and 7 and supplementary fig. S15, Supplementary Material online). These results indicate that while the residue configurations derived from the Metazoa–Fungi common ancestor (A1–A3) were able to catalyse p53 deacetylation, this ability was lost during the Fungal SIRT1 evolution (F1 and F3). Finally, the analysis of M1 and M3 variants, generated based on the ancestral sequences of the Metazoan sirtuins (Anc164), indicates, as expected, similar activity with p53 as WT hSIRT1 (figs. 6 and 7). Overall, these results suggest that the active-site configuration of the Metazoa–Fungi common ancestor exhibited multispecificity towards both histone and p53-like substrates. We suggest that this multispecificity trait was maintained during Metazoan SIRT1 evolution but was lost during Fungi SIRT1 evolution as these enzymes became more specialized.

## Discussion

Many enzymes that catalyze the addition or removal of diverse types of PTMs on different protein targets are multispecific. This phenomenon is extremely hard to decipher at the molecular level and remains enigmatic for many such enzymes (Beltrao et al. 2013). This study on hSIRT1, being one of the most prominent examples of multispecific hub proteins, addresses fundamental questions regarding the molecular basis and evolution of multispecificity in these enzymes. Using a combined bioinformatics-experimental approach, we identified and tested substitutions in SIRT1 that arose during the evolution of these enzymes in Eukaryota. The examination of different hSIRT1 variants with substrates that are either ubiquitous (e.g., histones) or only found in certain evolutionary branches of Eukaryota (e.g., p53) enabled the identification of a set of residues in hSIRT1, located near the entrance to the active site, that are essential for multispecificity, thereby shedding new light on the molecular determinants of this phenomenon.

hSIRT1 is an excellent model system to study multispecificity in PTM catalyzing enzymes due to its deacetylation activity toward a large number of proteins (Lavu et al. 2008). A recent study that examined the deacetylation profile of all human SIRT1-7 enzyme with 6,802 different acetylated peptides, representing the human acetylome, revealed that thousands of acetyl-lysine sites are targeted by these enzymes (Rauh et al. 2013). However, despite extensive studies, little is known regarding the residues in hSIRT1 that enable the high degree of multispecificity. Several studies on the specificity of hSIRT1, using different types of peptide arrays (Blander et al. 2005; Garske and Denu 2006), revealed no clear consensus sequence for acetyl-lysine recognition. In contrast to the multispecificity of hSIRT1, the substrate repertoire analysis of the ySir2 ortholog revealed specificity toward conserved histone acetyl-lysine residues (Bheda et al. 2012). This observation suggests that multispecificity in SIRT1 can be acquired or lost through natural evolution from a common ancestor.

The identification of semiconserved residues in the vicinity of the hSIRT1 active site allowed us to generate and focus on a subset of variants (V3/V4/V5), containing 2 or 4 substitutions at the vicinity of hSIRT1’s active site (figs. 1 and 2). Our findings that V3 and V5 exhibit high activity toward conserved histone substrates but a significant loss of activity toward acetylated p53 (figs. 4 and 5) suggest that the amino acids R446 and L450 in hSIRT1 are essential for p53 deacetylation activity (fig. 1). ASR followed by experimental examination of hSIRT1 mutants containing ASR based substitutions (fig. 6) revealed the dynamics of SIRT1-p53 recognition through evolution. Our findings that variants containing mutations derived from Fungal-Metazoa SIRT1 common ancestor exhibit activity with p53, suggest that this common ancestor most likely exhibited multispecificity. It suggests that evolution of multispecificity requires continual selective pressure via the presence of certain substrates. Thus, the absence of p53 in Fungi may alleviate selective pressure to maintain SIRT1 activity with p53 and this function was lost during mutational drift leading to higher specificity of fungal sirtuins. In contrast, in Metazoa there had been a selective pressure to maintain activity with p53. Interestingly, a recent evolutionary analysis of p53 indicated the existence of p53 homologs in Holozoa (Bartas et al. 2020). The Holozoa clade includes unicellular organisms and Metazoa but is distinct from Fungi. This analysis is consistent with our results and suggests the possibility that an ancestral p53-like protein was indeed present in the metazoan-fungi common ancestor and interacted with the SIRT1 ancestor. Since the fixation of K382 in p53 appeared only in vertebrates (Joerger et al. 2014) (fig. 1) it is possible that SIRT1 multispecificity preceded the appearance of p53 K382Ac site.

Function and evolution of promiscuous enzymes that catalyze the chemical transformation of native and nonnative substrates has been extensively studied (Babtie et al. 2010; Khersonsky and Tawfik 2010). Directed evolution of these enzymes revealed the robustness of the native enzyme activity to mutations that dramatically affect the promiscuous, nonnative activities (Aharoni et al. 2005). By analogy to these studies, it is tempting to speculate that the activity of multispecific enzymes, such as SIRT1, toward the most ancient, conserved, and ubiquitous substrates (e.g., histones) is robust to amino acid substitutions that more easily lead to reduced activity with more recently evolved substrates (e.g., p53). However, additional studies with other multispecific enzymes are required to thoroughly examine this hypothesis.

Overall, our study has allowed the identification of residues that are critical for SIRT1 multispecificity. For a more comprehensive analysis of SIRT1 multispecificity, analysis of additional SIRT1 substrates is required. In addition, future work should investigate the molecular basis for SIRT1 multispecificity via structural analysis of WT and mutant SIRT1 in complex with different protein substrates. Although structures of hSIRT1 in complex with peptides derived from p53 (Cao et al. 2015) have been solved, the future structural elucidation of SIRT1 in complex with full-length acetylated protein substrates (e.g., p53 or H4) could reveal in more detail exactly how 446, 450, 417, 424 residues in the vicinity of the SIRT1 active site interact with SIRT1 substrates. These studies could also reveal the importance of SIRT1 structural dynamics and conformational changes to substrate recognition. Finally, our approach can be further utilized for the examination of a variety of other multispecific PTM catalyzing enzymes including kinases/phosphatases, methyl-transferases/demethylases, and ubiquitin-ligases/deubiquitinases to study the molecular basis for their multispecificity and shed new light on the evolution of enzyme-substrate recognition in these diverse and important systems.

## Materials and Methods

### Sirtuin-Superfamily Wide SSN and Phylogenetic Profiles of hSIRT1 Substrates

The UniProtKB (UniProt Consortium 2013) and NCBI (NCBI Resource Coordinators et al. 2018) sequence databases were searched for hits of sequence patterns associated with sirtuin proteins. Signatures from InterPro (Finn et al. 2017) (IPR026590, IPR026591, IPR003000, IPR017328, IPR026587, IPR027546, and IPR028628) and Pfam (Punta et al. 2012) (PF02146, PF13289) yielded 10,273 unique sequences with pattern matches. The Structure-Function Linkage database tools (Barber and Babbitt 2012; Akiva et al. 2014) were then used to generate a representative SSN, as described before (Atkinson et al. 2009; Akiva et al. 2017). For phylogenetic profiles of hSIRT1 substrates, we complied a set of organisms that sample the phylogenetic tree of eukaryotes and have fully sequenced genomes. For each organism, we combined EggNog (Huerta-Cepas et al. 2016) and Inparanoid (Sonnhammer and Östlund 2015) to find orthologs of hSIRT1 substrates. Details can be found in supplementary materials and methods, Supplementary Material online.

### Cloning, Expression, and Purification of SIRT1 Mutants in *E. coli*

The p38 plasmid containing hSIRT1 (UniProt Q96EB6) gene fused N-terminal 6xHis tag was obtained as a kind gift from Haim Cohen lab, the Bar-Ilan University, Israel. The p38 was modified by deleting one of the two *Kpn*I restriction sites to generate the p38d plasmid followed by cloning of SIRT1 DAC domain containing the different mutations. Plasmids containing the WT and mutant hSIRT1 genes were transformed and expressed in Rosseta 2 *E. coli* competent cells by IPTG induction. hSIRT1 variants were purified by Ni-NTA purification. The purity of the proteins was assessed by SDS-PAGE on 10% gel and protein concentration was measured by the Bradford method. Details can be found in supplementary materials and methods, Supplementary Material online.

### FDL and Ammonia-Coupled Assays for hSIRT1 Variants

The activities of the mutants were measured by FDL assay using protected *N*-acetyl lysine substrate conjugated to a 4-amino-7-methylcoumarin group (AMC) at the carboxyl terminus. The assay was performed based on previously described protocol (Wegener et al. 2003) with modifications. The ammonia-coupled assay was performed as previously described (Smith et al. 2009) with minor modifications. The ammonia assay kit (Sigma) and relevant acetylated peptides (Peptron) were used for preparing the reaction mix. The PNC1 plasmid was obtained as a kind gift from Jessica L. Will and Jorge C. Escalante-Semerena from the University of Georgia and the protein for the assay was purified as previously described (Garrity et al. 2007). The sequences of all peptides used in this study are shown in supplementary table S4, Supplementary Material online. Details can be found in supplementary materials and methods, Supplementary Material online.

### Native Histone and p53 Deacetylation Assays

Chromatin fractionation was performed as previously described (Gertman et al. 2018) with minor modifications. The full-length proteins were expressed in bacteria that incorporated the non-natural amino acid (acK) by expanding the codon usage. The p53 plasmid with the stop codon in position 382 was cloned to the pCDF Duet plasmid and purified as previously described (Arbely et al. 2011). Detailed description of activity assays for native histones and acetylated p53 protein can be found in supplementary materials and methods, Supplementary Material online.

### Coevolution Analysis and ASR of SIRT1

Coevolution analysis was performed on an automatically compiled >6,000 sequence MSA of the SIRT1 DAC sequences using the GREMLIN (Balakrishnan et al. 2011) online server (http://gremlin.bakerlab.org/). For construction of a maximum likelihood tree, an EFI-EST enzyme similarity networks (Gerlt et al. 2015) were used to collect sequence orthologous to hSIRT1, using the *Xenopus laevis* SIRT1 sequence as seed. Detailed description of phylogenetic tree construction and ASR can be found in supplementary materials and methods, Supplementary Material online.

## Supplementary Material

Supplementary data are available at *Molecular Biology and Evolution* online.

## Supplementary Material

msaa244_Supplementary_Data
