# Supplementary material for: Human SIRT1 Multispecificity Is Modulated by Active-Site Vicinity Substitutions during Natural Evolution
Source: Mol Biol Evol. 2020 Sep 21;38(2):545–56. doi: 10.1093/molbev/msaa244 (PMC7826192; doi:10.1093/molbev/msaa244)
Supplement: msaa244_Supplementary_Data [file msaa244_supplementary_data.docx]

**Supplementary Information**

**Human SIRT1 multi-specificity is modulated by active-site vicinity substitutions during natural evolution**

Adi Hendler^1,5^, Eyal Akiva^2,5^, Mahakaran Sandhu^3^, Dana Goldberg^1^, Eyal Arbely^4^, Colin J. Jackson^3^ and Amir Aharoni^1*^

^1^Department of Life Sciences and the National Institute for Biotechnology in the Negev, Ben-Gurion University of the Negev, Be’er Sheva 84105, Israel. ^2^Department of Bioengineering and Therapeutic Sciences, University of California, San Francisco, CA 94158, USA. ^3^Research School of Chemistry, Australian National University, Acton, 2601, ACT, Australia. ^4^Department Chemistry and the National Institute for Biotechnology in the Negev, Ben-Gurion University of the Negev, Be’er Sheva 84105, Israel. ^5^These authors contributed equally to this work

^*^ Correspondence should be addressed to A.A. ([aaharoni@bgu.ac.il](mailto:aaharoni@bgu.ac.il))

**Material and Methods**

**Collecting sirtuin sequences and generating a sirtuin-superfamily wide sequence similarity network**

The UniProtKB (UniProt Consortium 2013) and NCBI (NCBI Resource Coordinators et al. 2018) sequence databases were searched for hits of sequence patterns associated with sirtuin proteins. Signatures from InterPro (Finn et al. 2017) (IPR026590, IPR026591, IPR003000, IPR017328, IPR026587, IPR027546 and IPR028628) and Pfam (Punta et al. 2012) (PF02146, PF13289) yielded 10,273 unique sequences with pattern matches. The Structure-Function Linkage database tools (Barber and Babbitt 2012; Akiva et al. 2014) were then used to generate a representative SSN, as described before (Atkinson et al. 2009; Akiva et al. 2017). In this network, nodes represent sets of proteins that share >60% sequence identity as measured by Cd-hit (Li and Godzik 2006), and edges represent a mean *E*-value more significant than 1×10^-18^ between all pairwise *E*-value scores calculated between the sequences represented in each node. In the case of the SSN presented in **Fig. 3C**, each node represents one sequence. Identifying this cutoff was obtained by manual sampling of several edge inclusion thresholds until a reasonable reconciliation was achieved between distinct similarity clusters and representation of remote similarities between them. Networks in this paper are visualized by Cytoscape (Shannon et al. 2003) (organic layout).

**Phylogenetic profiles of hSIRT1 Substrates**

We complied a set of organism that sample the phylogenetic tree of eukaryotes and have fully-sequenced genomes. For each organism, we combined EggNog (Huerta-Cepas et al. 2016) and Inparanoid (Sonnhammer and Östlund 2015) to find orthologs of hSIRT1 substrates. These auto-generated lists of orthologs were further validated by finding best BLAST (Altschul et al. 1990) reciprocal hits between the human proteome and any of the eukaryotic proteomes (downloaded from UniProtKB (UniProt Consortium 2013) and EnsEMBL (Zerbino et al. 2018)). These results were manually examined and then cross-validated with literature-documented phylogenetic models for each substrate, whenever available. Fig. 1 includes a color-coded summary of the results: From white (no ortholog was detected) to dark red (highly conserved ortholog). Color intensity is proportional to the bit-score (as computed by BLAST), divided by the alignment length. To evaluate the conservation of putative acetyl-Lysine residues in the substrates, we aligned orthologs of each substrate using MAFFT (Katoh 2002) and focused on the segments that align with the human acetyl-Lysine. **Fig. 1** summarizes the results of these analysis by the letter “K” for each substrate and organism. Absence of the letter means that no Lysine was found in the relevant aligned segment.

**Identification of the specificity-determining residues**

The MSA of the DAC domain includes 268 positions. In order to focus on specific positions to mutate, we used the following criteria: (a) We predicted surface accessibility of positions based on human Sirt-1's crystal structure (PDB 4KXQ) and removed 114 positions that are clearly buried (ASA<25Å, as calculated by PyMOL). (b) We used the MSA to omit positions that have very low information content (for instance, positions that are universally conserved throughout the alignment). To this end, we calculated the Shannon entropy and omitted additional 93 positions for which the entropy value was lower than 1.0. We used the Shannon entropy cutoff of 1.0, since it is considered as the cutoff for variability. Specifically, any value <1.0 is considered as representing an MSA position that having low variability (Stewart et al. 1997) (c) To identify positions in which dramatic changes in amino acid properties are indicated, we examined the remaining 61 positions and removed an additional set of 24 positions in which the physicochemical characteristics of the amino acids in each position was conserved. Finally, the remaining 37 positions were reduced to eight positions (**Fig. 1**) via further visual inspection of the crystal-solved structure of human Sirt-1 and the main taxonomical branches mapped to the MSA. This analysis allowed the identification of residues in the Rossmann fold domain (positions 481 and 484), the active site region (positions 417, 424, 446 and 450) and the Zinc binding domain (positions 372 and 380) (**Figs. 1-2**).

**Generation of SIRT1 mutant plasmids**

The p38 plasmid containing hSIRT1 (UniProt Q96EB6) gene fused N-terminal 6xHis tag was obtained as a kind gift from Haim Cohen lab, the Bar-Ilan University, Israel. The p38 was modified by deleting one of the two KpnI restriction sites to generate the p38d plasmid. All further genetic manipulations were based on the p38d plasmid. For generating hSIRT1 mutants in the DAC domain, the mutants were cloned into p38d using KpnI and HindIII. The DAC domain mutants were obtained by a PCR reaction in several steps. The First step was performed to amplify the DAC domain in two fragments with a primer that includes the relevant mutation. The two fragments were purified using the GeneJET PCR extraction kit (Thermo), followed by assembly PCR and further amplification of the whole mutant DAC domain. The KOD polymerase (Merck) was used for all PCR amplifications. The resulting mutated DAC PCR fragments were incubated with DpnI (Thermo) for 30 min at 37°C followed by 10 min of heat inactivation at 80°C. The DAC mutants were further purified and cloned to the digested p38d vector using NEB builder Gibson mix (NEB). The V5 mutant was ordered as a synthetic gene, amplified by PCR and cloned as described above. The final product was transformed to NEB competent bacterial cells and plated on LB agar containing 100 µg/ml Ampicillin. Positive colonies were verified by PCR with dreamtaq polymerase (Thermo), followed by purification using NucleoSpin Plasmid EasyPure kit (Macherey-Nagel) and sequenced to verify the correct mutations.

**Expression and purification of SIRT1 mutants and PNC1 in *E. coli***

Plasmids containing the WT and mutant hSIRT1 genes were transformed into Rosseta 2 *E. coli* competent cells, grown in TB medium containing ampicillin and chloramphenicol at 37°C to an OD_600_ of 0.6 and induced with 0.8 mM IPTG followed by 16 hours of incubation at 16°C. Cells were lysed by sonication in lysis buffer containing 40 mM Tris-HCl pH 8, 200 mM NaCl, 10 mM MgCl_2_, 1:2000 EDTA free-protease inhibitor cocktail (Calbiochem) and Benzonaze (Mercury). Cell debris were removed by centrifugation at 10,000g at 4°C for 30 min. The supernatant was loaded onto pre-equilibrated nickel beads, washed with wash buffer containing 40 mM Tris-HCl pH 8, 200 mM NaCl, 10 mM MgCl_2_ and 20 mM imidazole and eluted with elution buffer (similar to wash buffer but containing 500 mM imidazole). The eluted protein was dialyzed with activity buffer containing 25 mM Tris-HCl pH 8, 200 mM NaCl, 1mM MgCl_2_ and 2.5mM DTT, for imidazole removal. The purity of the proteins was assessed by SDS-PAGE on 10% gel and protein concentration was measured by the Bradford method using Bovine Serum Albumin (BSA) as the standard. The PNC1 plasmid was obtained as a kind gift from Jessica L. will and Jorge C. Escalante-Semerena from the University of Georgia and the protein was purified as previously described(Garrity et al. 2007).

**Fluor de lys (FDL) activity assay for WT and SIRT1 mutants**

The activities of the mutants were measured by FDL assay using protected acetylated lysine substrate conjugated to a 4-amino-7-methylcoumarin group (AMC) at the carboxyl terminus. The assay was performed using the following protocol: 30 µl of the hSIRT1 WT or mutants at 1.5 µM were added to 20 µl of the reaction mix containing 12.5 mM NAD+, 1.25 mM AcLys-AMC in 50 mM Tris-HCl pH 8.0, 137 mM NaCl, 2.7 mM KCl, 1 mM MgCl_2_ and BSA 1 mg/ml (FDL buffer). The mixture was incubated in black 96 well plates (Greiner) covered with aluminum foil on ice. The reaction was stopped at different time points by adding 50 µl developer solutions (3 mg/ml Trypsin, 0.05 mM HCl in FDL buffer with BSA). After adding the developer, the plate was incubated in 37°C for 40 min and fluorescence intensities were measured using Infinite M200 plate reader (Tecan).

**Ammonia coupled assay with acetylated peptides**

The ammonia coupled assay was performed as previously described (Smith et al. 2009) with minor modifications. The ammonia assay kit (Sigma) and relevant acetylated peptides (Peptron) were used for preparing the reaction mix. The sequences of all peptides used in this study are shown in **Table S2**. This mix contained 75 µl activity buffer, 1 µl of L-Glutamate Dehydrogenase (Ammonia kit, Sigma), 2 µl of 150 mM NAD+, 2 µl of 1 µM purified PNC1, and 16 µl of 1.5 µM hSIRT1. Acetylated peptides were added at different concentration and the volume was completed to 100 µl with DDW. The reactions were measured at 320 nm for 30 min at 30°C using Infinite M200 plate reader.

**Chromatin fractionation and histone deacetylation assay**

Chromatin fractionation and deacetylation assay on histones was performed as previously described (Gertman et al. 2018). The deacetylation assay was performed using 70 µl of chromatin fraction, 20 µl 125 mM NAD+, 30 µl of activity buffer (30 mM Tris-HCl pH 8, 4 Mm MgCl_2_ and 1 mM DTT) in the presence of 40 µl of 1.5µM purified hSIRT1. The reaction was stopped at different time point of 0, 15 sec, 30 sec, 45 sec, 1 min, 1.5 min, 2 min and 2.5 min. To detect the decrease in acetylation of H4K16Ac, the samples were resolved on 15% SDS-PAGE gel and transferred with Tran-Blot Turbo Transfer Pack (Bio Rad) using Trans Blot Turbo Transfer System. The membrane was then probed with several antibodies: Rabbit α-H4 (ab10158), Rabbit α-H4K16 (ab109463) (abcam) followed by a secondary antibody goat α-rabbit. For the analysis of hSIRT1 levels, α-his tag conjugated to HRP was used. Western blot bands were quantified using Image J program.

**Purification and deacetylation assay on native p53**

The full length proteins were expressed in bacteria that incorporated the non-natural amino acid (AcK) by expending the codon usage. The p53 plasmid with the stop codon in the position 382 was cloned to the pCDF Duet plasmid and purified as previously described (Arbely et al. 2011). In the deacetylation assay we used 140µl of 4µM of the full length substrate, 80µl of 0.8µM of purified hSIRT1 or mutants and 100µl deacetylation buffer (25 mM Tris, pH 8.0, 137 mM NaCl, 2.7 mM KCl, 1 mM MgCl_2_, 1 mM NAD+) (Knyphausen et al. 2016). The reaction was the incubated at room temperature and samples were removed at different time points of 0 min, 15 min, 30 min, 45 min, 60 min, 75 min, 90 min and 120 min. All the samples were analysed by western blot with several antibodies: Rabbit α-p53 (#9282), Rabbit α-p53 ac-K382 (#2525) (Cell Signaling) followed by goat α-rabbit or α-mouse antibody conjugated to HRP. Analysis of hSIRT1 levels and band quantification were performed as described above.

**Coevolution analysis, maximum-likelihood phylogenetic inference and ancestral sequence reconstruction**

Coevolution analysis was performed on an automatically compiled >6000 sequence MSA of the SIRT1 DAC (using the hSIRT-1 DAC sequence as the initial search sequence) using the Generative REgularised ModeLs of ProteiNs (GREMLIN) (Balakrishnan et al. 2011) online server (<http://gremlin.bakerlab.org/>). The pairwise coevolution matrix output by GREMLIN was arranged into a more informative network representation using Cytoscape(Shannon et al. 2003), with an edge cut-off at a GREMLIN raw co-evolution score of ~1.28 to obtain well-delineated and mutationally viable modules of co-evolving residues.

For construction of a maximum likelihood tree, an EFI-EST enzyme similarity network (Gerlt et al. 2015) were used to collect sequences orthologous to hSIRT1, using the *Xenopus laevis* SIRT1 sequence as seed. Using incremental percent identity edge cutoffs in Cytoscape (Shannon et al. 2003), clusters delineated by sirtuin subfamily were obtained. The SIRT1 cluster was identified by locating the human SIRT1 sequence (Q96EB6). We explored this cluster to ensure that the sequences found were orthologous to SIRT1.

Thus, ~ 400 sequences of hSIRT1 likely orthologues were collected from a number of Eukaryotic kingdoms. Incomplete fragment sequences were purged by manual inspection. Sequences with greater than 90% sequence homology were clustered and represented by a single sequence with CD-HIT (Li and Godzik 2006) thereby removing redundant sequences from the sequence set. The PROMALS3D alignment program (Pei et al. 2008) was used to align these sequences. The alignment was extensively inspected and the highly divergent N- and C- terminal domains removed. Another round of CD-HIT was followed by PROMALS3D alignment on the truncated sequences. Gaps and alignment errors were corrected through careful manual inspection of the resultant alignment. In particular, highly divergent and un-alignable insertions were observed in most non-Chordate phyla in the zinc-binding domain; these were removed except in the Chordates, where they were deemed to be truly homologous under parsimony criteria. The sequence set was manually enriched with chordate sequences using profile alignment with HMMer (Johnson et al. 2010). Alignments were benchmarked *via* structural alignment of the orthologs ScSIR2 (PDB:4IAO) and hSIRT1 (PDB:4KXQ;4IG9).

Phylogenies were calculated using IQ-TREE (Nguyen et al. 2015) a final alignment of 151 sequences, using ModelFinder (Kalyaanamoorthy et al. 2017) to find the best-fit evolutionary model and the UltraFast (Minh et al. 2013) bootstrap method to evaluate non-parametric bootstrap branch supports using 1000 pseudoreplicate trees. For inference of constrained-topology phylogenies, the constraint tree topology was manually constructed in accord with the prevailing species trees for the relevant taxa (dos Reis et al. 2015; Spatafora et al. 2016). All statistical topology tests were performed in IQ-TREE using 10,000 pseudoreplicate trees. Ancestral sequence reconstruction was performed using the CodeML program in PAML (Yang 2007), using the LG evolutionary model (Le and Gascuel 2008).

**
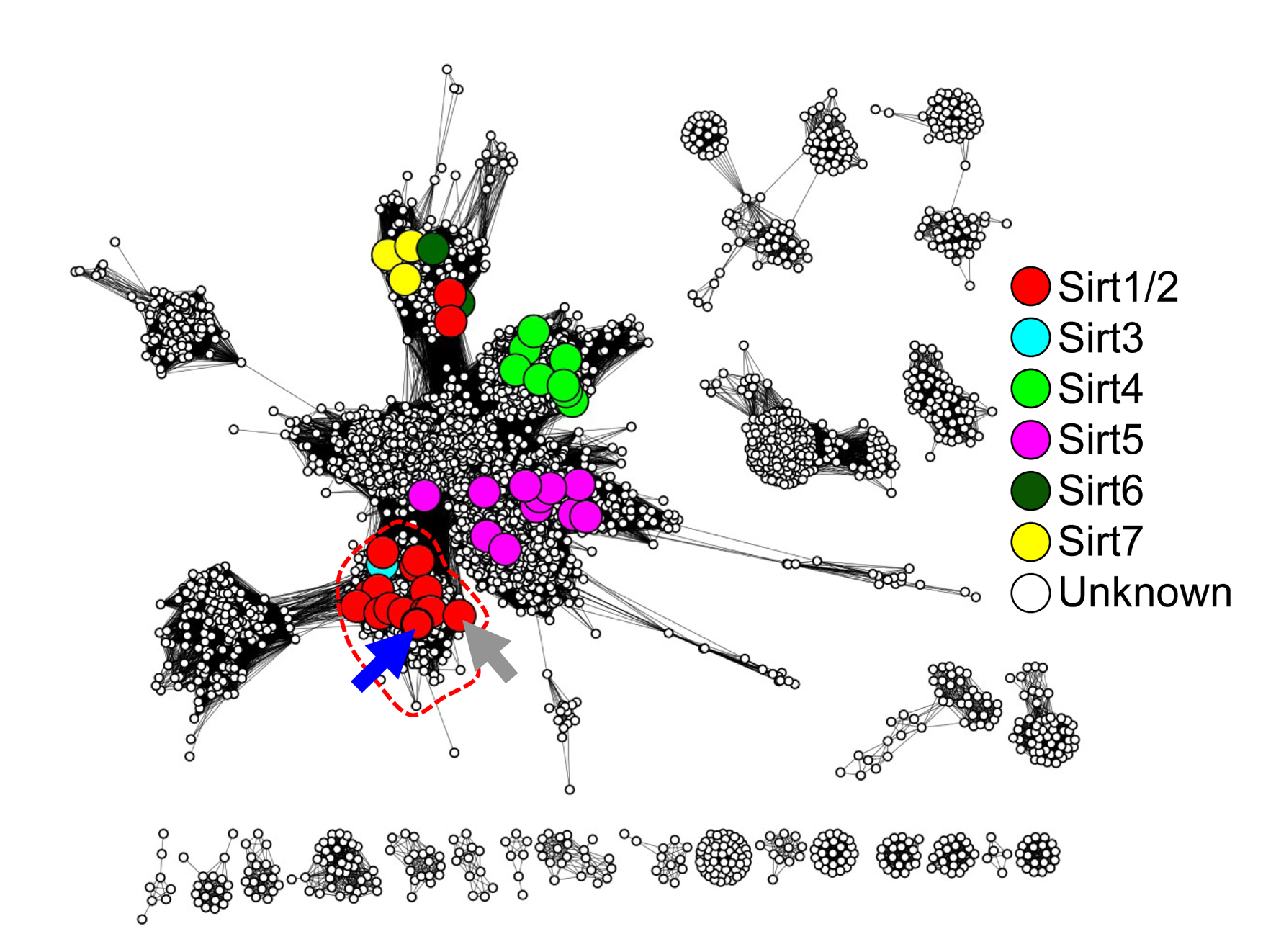
**

**Fig. S1: A representative sequence similarity network of the Sirtuin superfamily.** Nodes represent proteins that share 80% sequence identity, and edges represent average E-Value score below 1*10^-35^. Colored, large nodes represent sequence sets that include at least one experimentally verified member of the Sirtuin superfamily. The graphical layout used to represent the network creates separated clusters, each representing sequences that are more similar to each other than to any other sequences. The correspondence between similarity-based clustering and the canonical Sirtun families is clearly observed. The non-colored nodes represent uncharacterized sirtuins, hinting at yet unexplored sequence space of the superfamily. A blue arrow points at hSIRT1, and a grey arrow points at ySir2 . Note that Sirt1/2 are colored in red to clearly resolve the confusing, yet widely used naming conventions for these proteins. The upper cluster include “Sirtuin-1” superfamily members that belong to plants. The closest homologs of plant SIRT1s are SIRT6 proteins in non-plant organisms, exemplifying the problematic naming system of Sirtuin proteins.


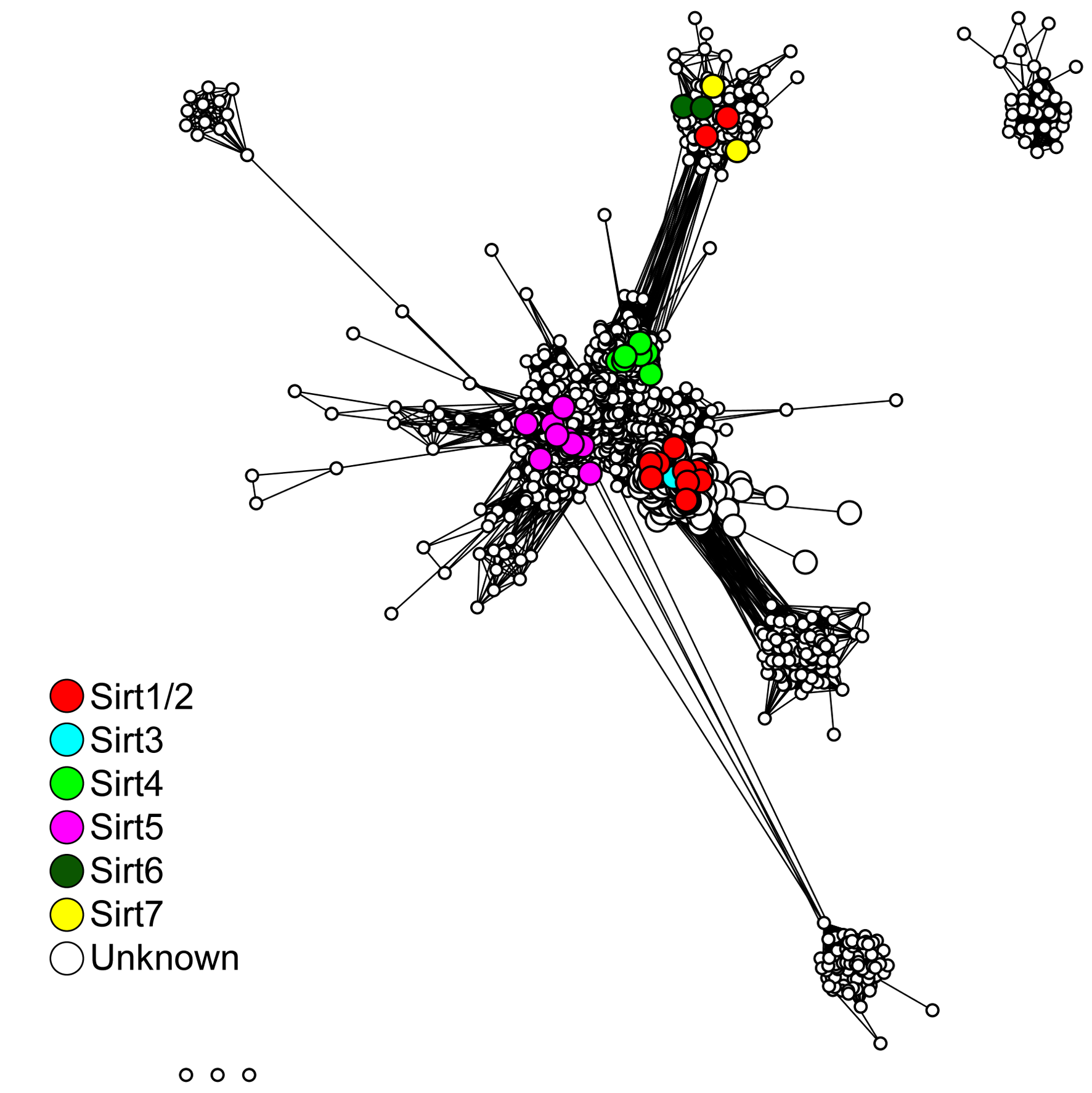


**Fig. S2:** SSN of the Sirtuin family based on the sequences of the DAC domain. The Pfam sequence pattern (PF13289) was used to to identify the boundaries of the Sirtuin DAC domain within all the Sirtuin superfamily members, and these protein segments were used to generate a new sequence similarity network (average E-Value score below 1*10^-34^). We then highlighted (see red nodes) all the sequences that are considered as the Sirtuin-1 family, as shown in **Fig. S1**. The topology of this network shows clear separation of the Sirtuin-1 family from other members of the Sirtuin superfamily.


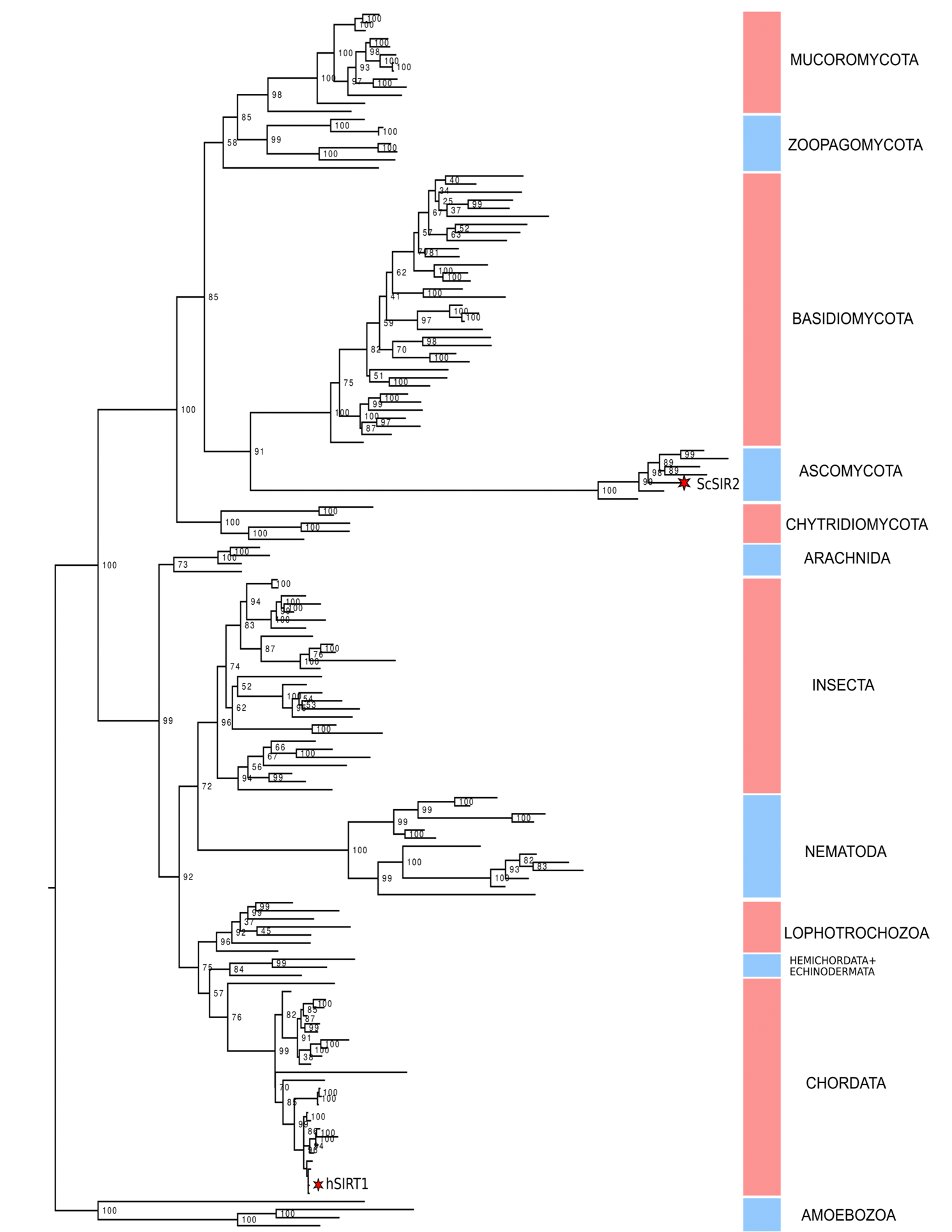


**Fig. S3: Representative tree of eukaryotic orthologs of SIRT1.** The maximum-likelihood phylogeny based on a 151 sequence alignment of SIRT1 family inferred with IQ-TREE using the LG+I+G4 evolutionary model. Bootstrap branch supports from 1000 pseudoreplicate trees asevaluated with the UltraFast bootstrap method are indicated at nodes. The hSIRT1 and *Saccharomyces cerevisiae* Sir2 (ScSIR2) are labeled in red.


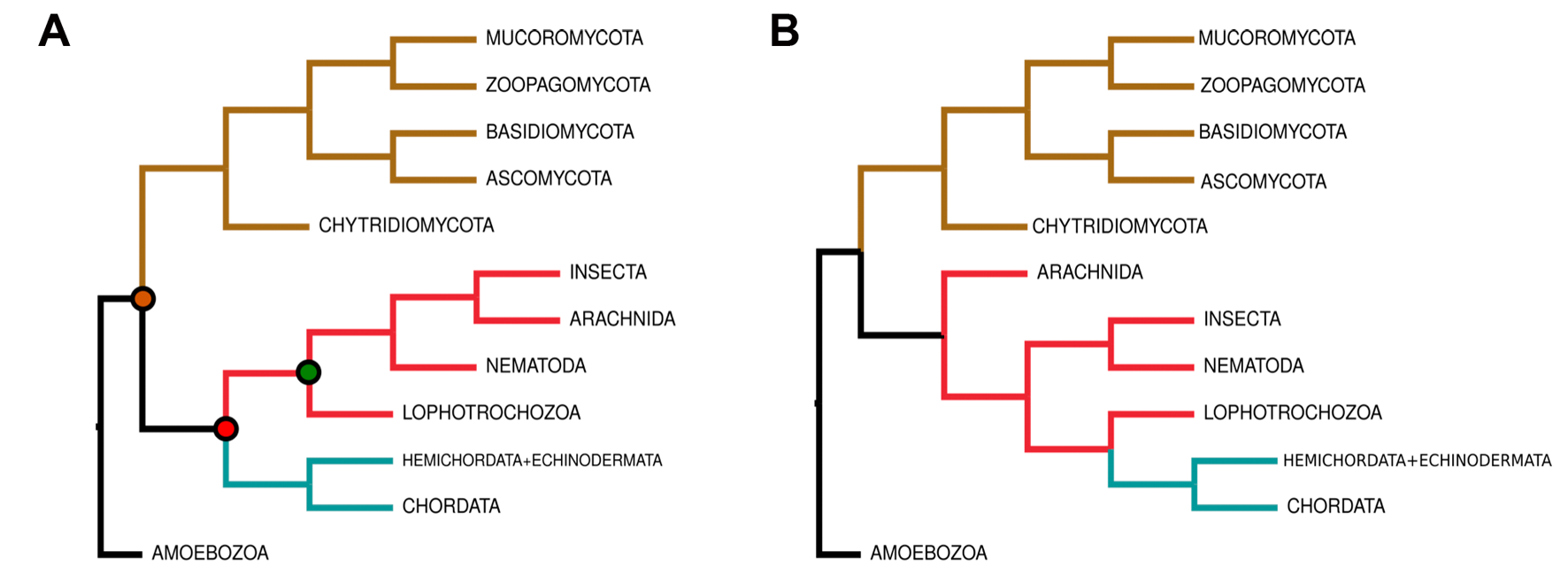


**Fig. S4:** (**A**) The established species cladogram, indicating relationships between taxonomic kingdoms (e.g. Amoebozoa), phyla (e.g. Ascomycota) and classes (e.g. Insecta) as appropriate for clarity. (**B**) Cladogram of the relationships observed according to the SIRT1 Maximum likelihood tree. Blue branches indicate deuterostome clades; red branches indicate protostome clades; brown branches indicate fungal clades. Notably, in **B**, the deuterostomes are placed as sub-clade of the protostomes, and the Arachnida are placed as the basal Metazoan clade. Their positions are different from their respective position in the tree of life.

**Discussion of the causes of tree discordance:** Artefactual causes of gene-tree discordance can include (1) low number of informative sites, (2) the presence of paralogs, (3) long-branch attraction (LBA), or (4) poor choice of evolutionary model. With respect to (3), LBA is the tendency of highly divergent (i.e. rapidly evolving) clades to group together in a phylogeny, regardless of their true phylogenetic relationship. Rapidly evolving clades accumulate many mutations that segregate them from the remainder of the tree. Due to the limited number of possible states (20 amino acids) at each homologous residue position, two phylogenetically unrelated clades can acquire similar mutations by chance, leading to their being identified as a monophyletic group (Bergsten 2005; Yang 2014) (this is distinct from convergent evolution, where similar mutations are not the product of chance but of selection). With respect to (4), if an alignment contains a high proportion of sites that are invariable, or conversely, a high proportion of sites variable to the point of being random (or both), the alignment will not be very informative regarding the true relationships among taxa, and a poor fit between the sequence alignment data and the evolutionary model chosen to describe it will result in artefactual topologies (Yang 2014) .In the case of this phylogeny, hemiplasy or convergent evolution between the the Deuterostomes and Lophotrochozoa is unlikely and HGT between animals is unheard of. LBA is unlikely given that branch lengths are generally short (except in the Ascomycota). Our method of using sequence similarity networks is robust to paralogs. Thus, the most probable explanation is that the discordance is an artefact of the nature of sequence data, probably due to the low number of informative sites.


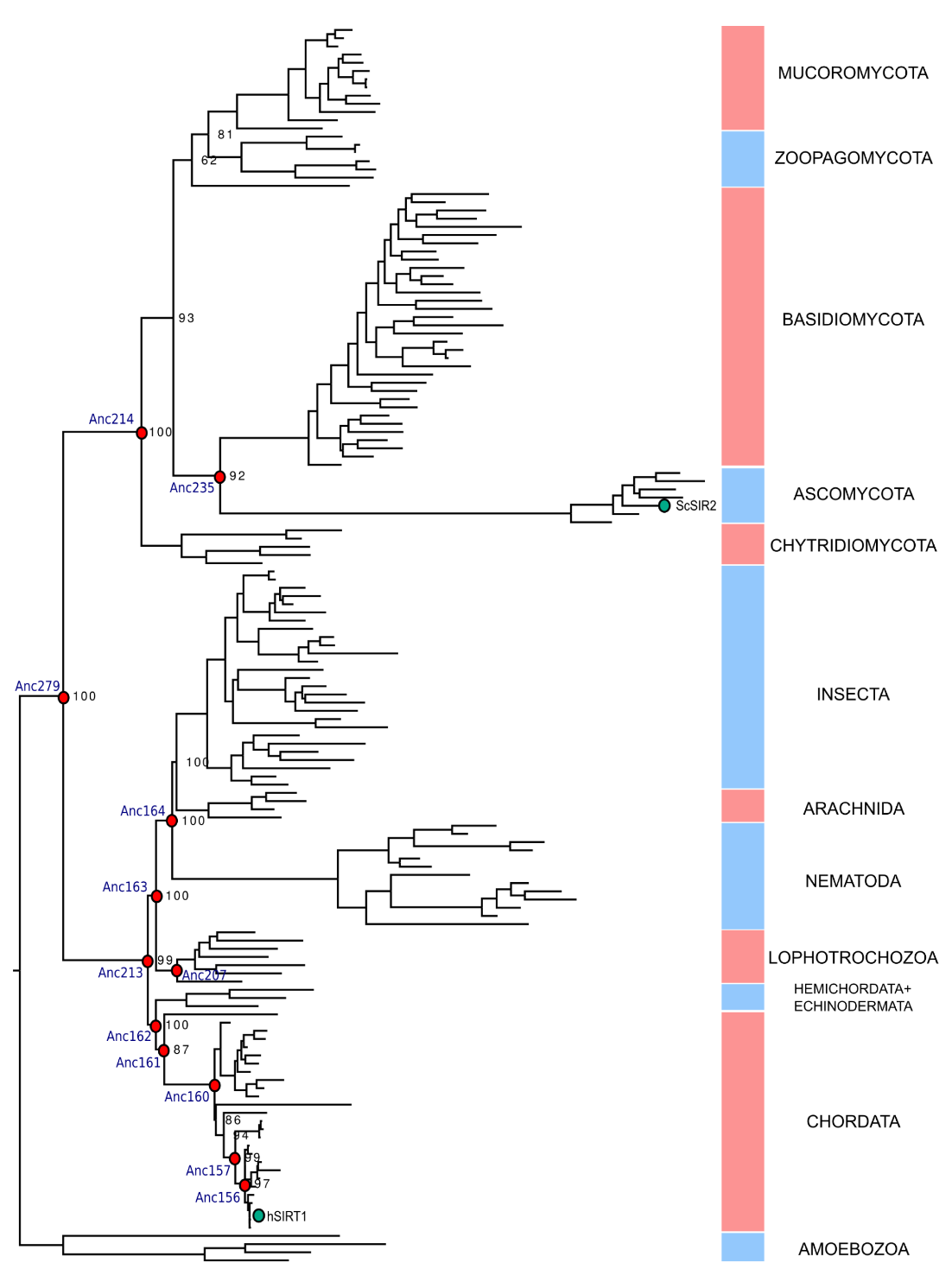


**Fig. S5:** The constrained phylogeny based on a 151 sequence alignment of SIRT1 family inferred with IQ-TREE using the LG+I+G4 evolutionary model. Reconstructed nodes are labelled in red. The hSIRT1 and *Saccharomyces cerevisiae* Sir2 (ScSIR2) are labeled in green.


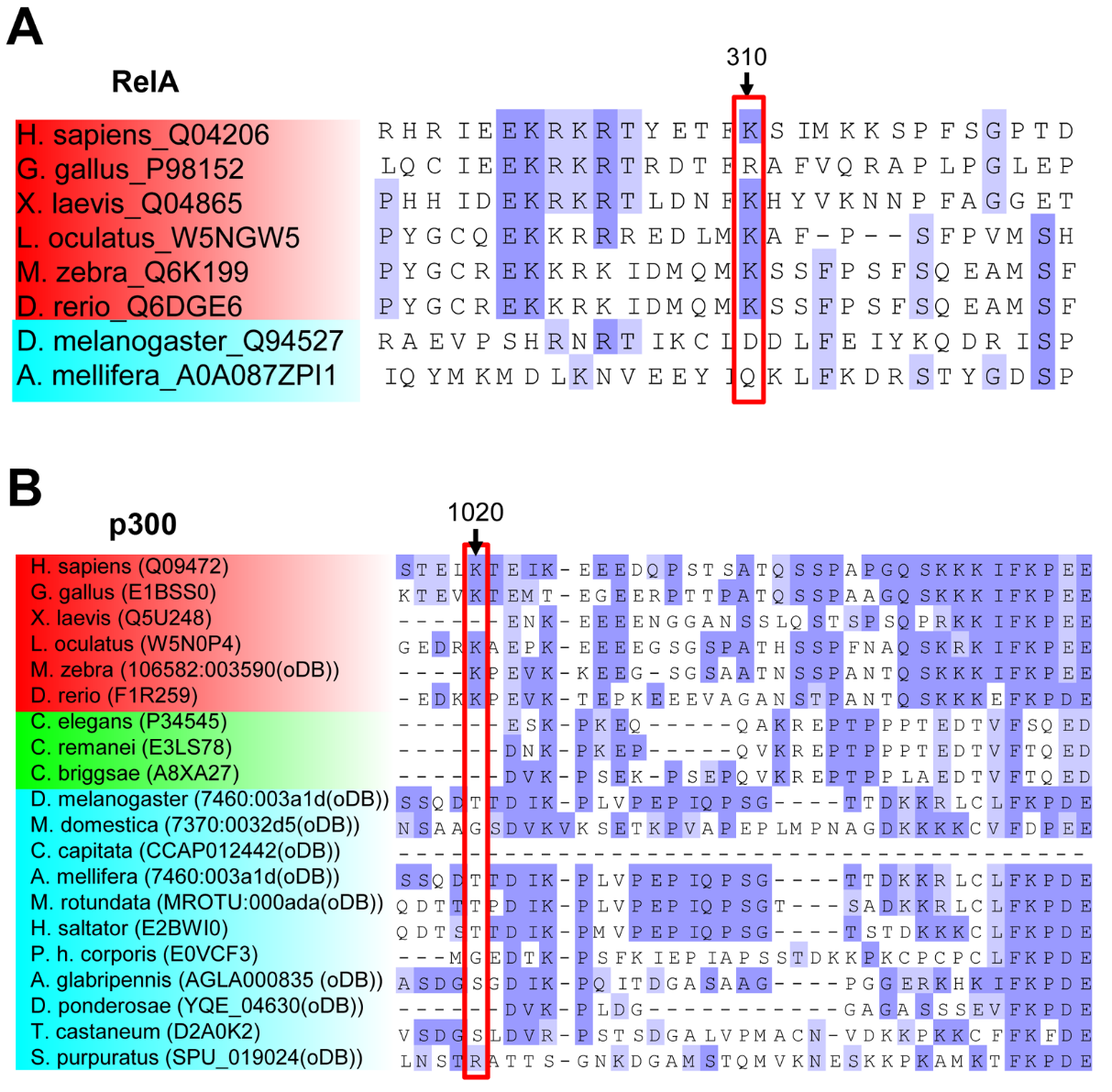


**Fig. S6**: **Sequence alignment of RelA/p65 and p300 orthologs emphasizing the conservation of position 310 and 1020 in human RelA and human p300, respectively**. Orthologs are based on consensus between diverse databases (OrthoDB, InParanoid, EggNog and EnsEMBL) and (Yuan and Giordano 2002; Wang et al. 2006). Protein identifiers are UniProtKB accession numbers or OrthoDB identifiers.

**
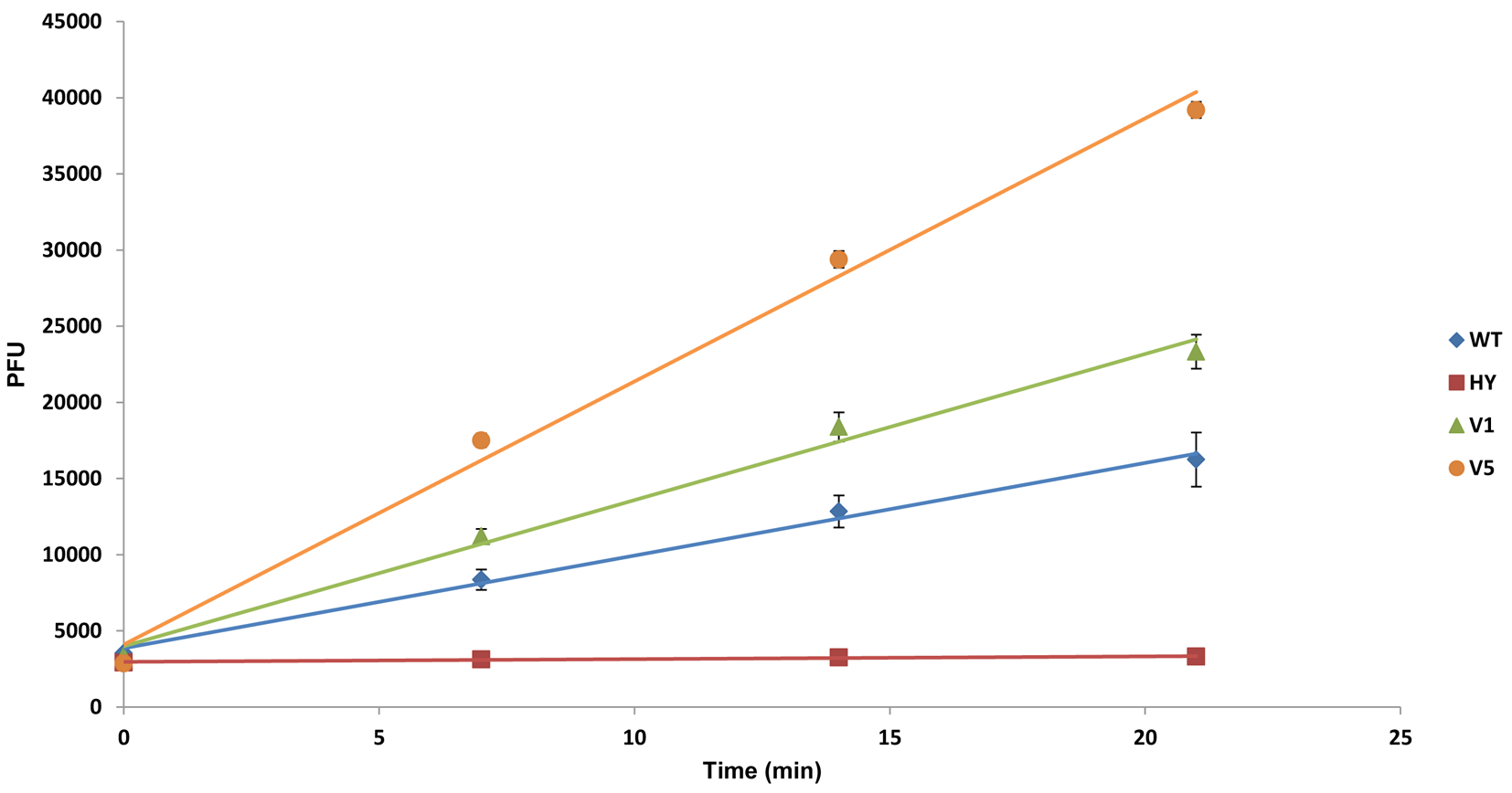
**

**Fig. S7: Representative Fluor De lyse (FDL) analysis of hSIRT1 mutants with protected acetyl-lysine-AMC substrate.** Fluorescent signal was measured at different time points after stopping the catalytic deacetylation reaction. Initial rates were calculated by fitting the data to linear equation and comparing to the slope obtained for the WT (**Fig. 3B**). The inactive HY hSIRT1 mutant containing the H363Y mutation serves as a negative control. All measurements were performed in triplicates and the standard deviation from the mean is shown.


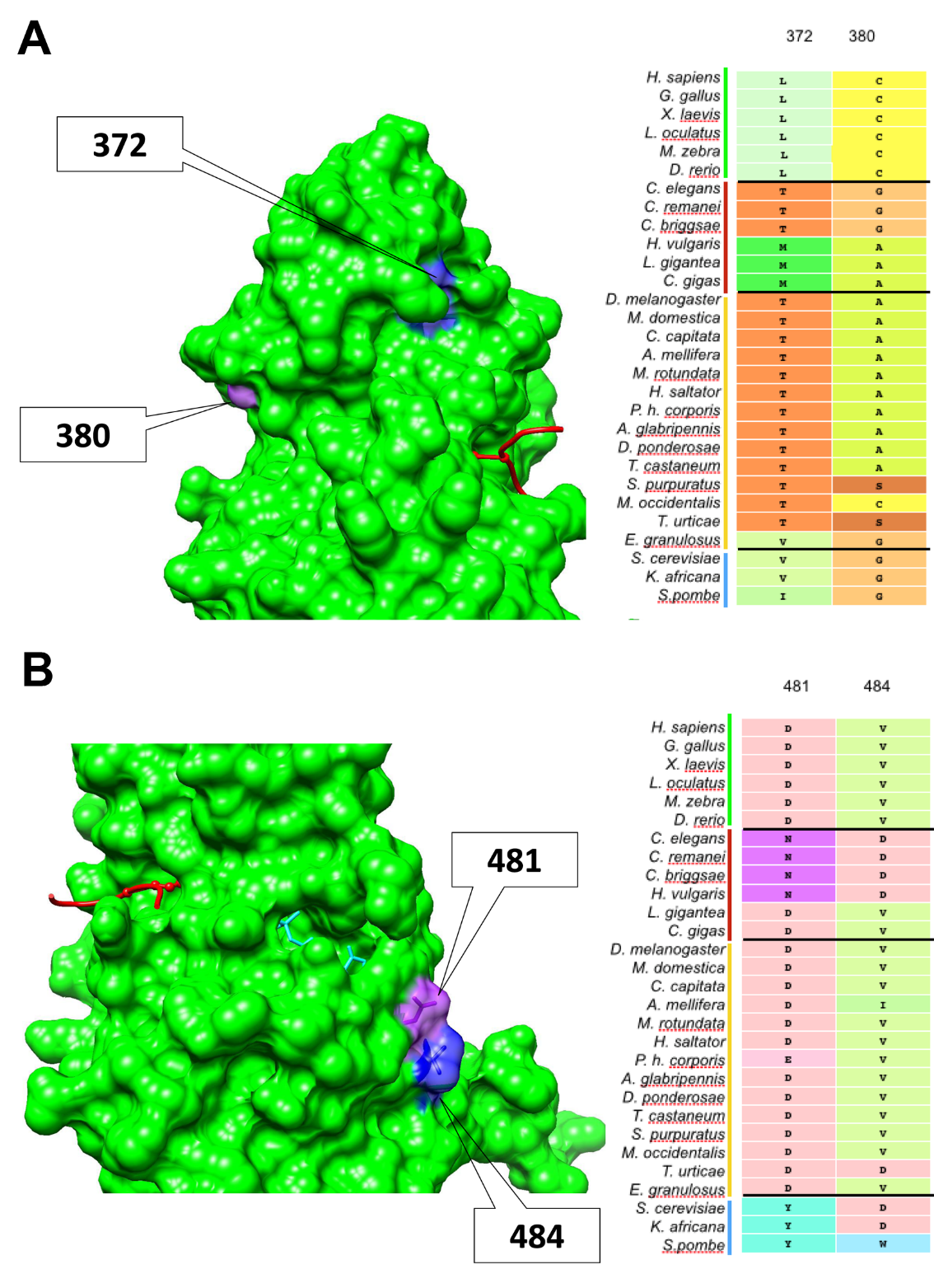


**Fig. S8: Evolutionary analysis of specific positions in SIRT1.** (**A**) Analysis of positions 372 and 380 located at the Zinc-binding domain. Left: Surface representation of hSIRT1 structure with positions 372 and 380 highlighted. Right: Sequence alignment of SIRT1 orthologs showing amino-acid identities at these positions. A correspondence can be observed between the amino acid in these positions and main taxonomic branches (**B**) Analysis of positions 481 and 484 located at the Rossmann fold domain. Left: Surface representation of hSIRT1 structure with positions 481 and 484 highlighted. Right: Sequence alignment SIRT1 orthologues showing amino-acid identities at these positions.

**
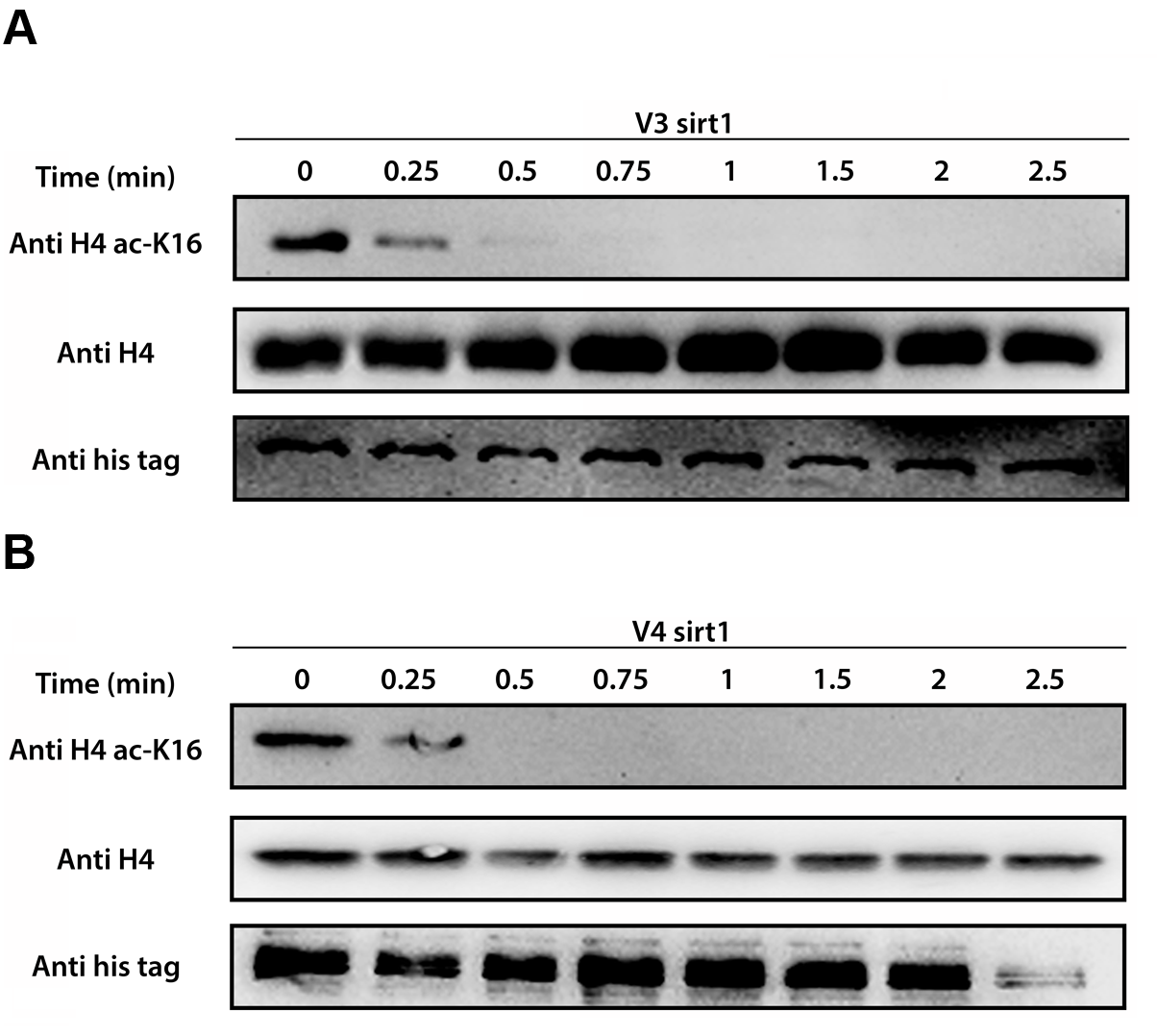
**

**Fig. S9**: **High V3 and V4 deacetylation activity toward native H4K16Ac.** Western blot kinetic analysis of V3 (**A**) and V4 (**B**) activity toward H4K16Ac in the context of native histones. The H4K16Ac, H4 and hSIRT1 were detected using anti-H4K16Ac antibody, anti-H4 antibody and anti-6xHis antibody, respectively, as described in Methods section.


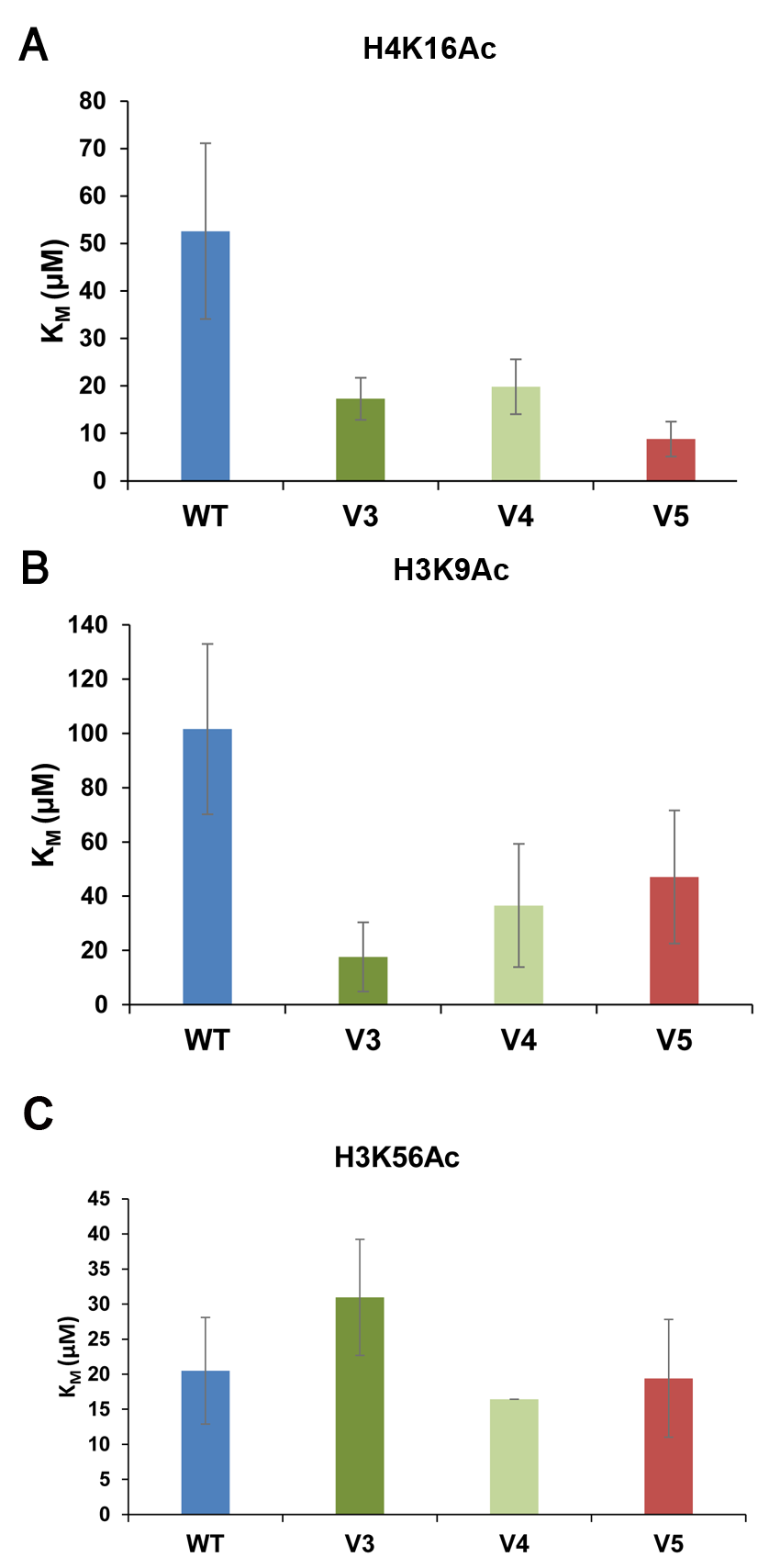


**Fig. S10**: **The activity of V3-V5 toward H4K16Ac, H3K9Ac and H5K56Ac peptides is maintained**. The K_M_ values of the WT and V3-V5 for these peptides were measured by the continuous assay (Smith et al. 2009). Full MM curves are shown in **Fig. S11**.

**
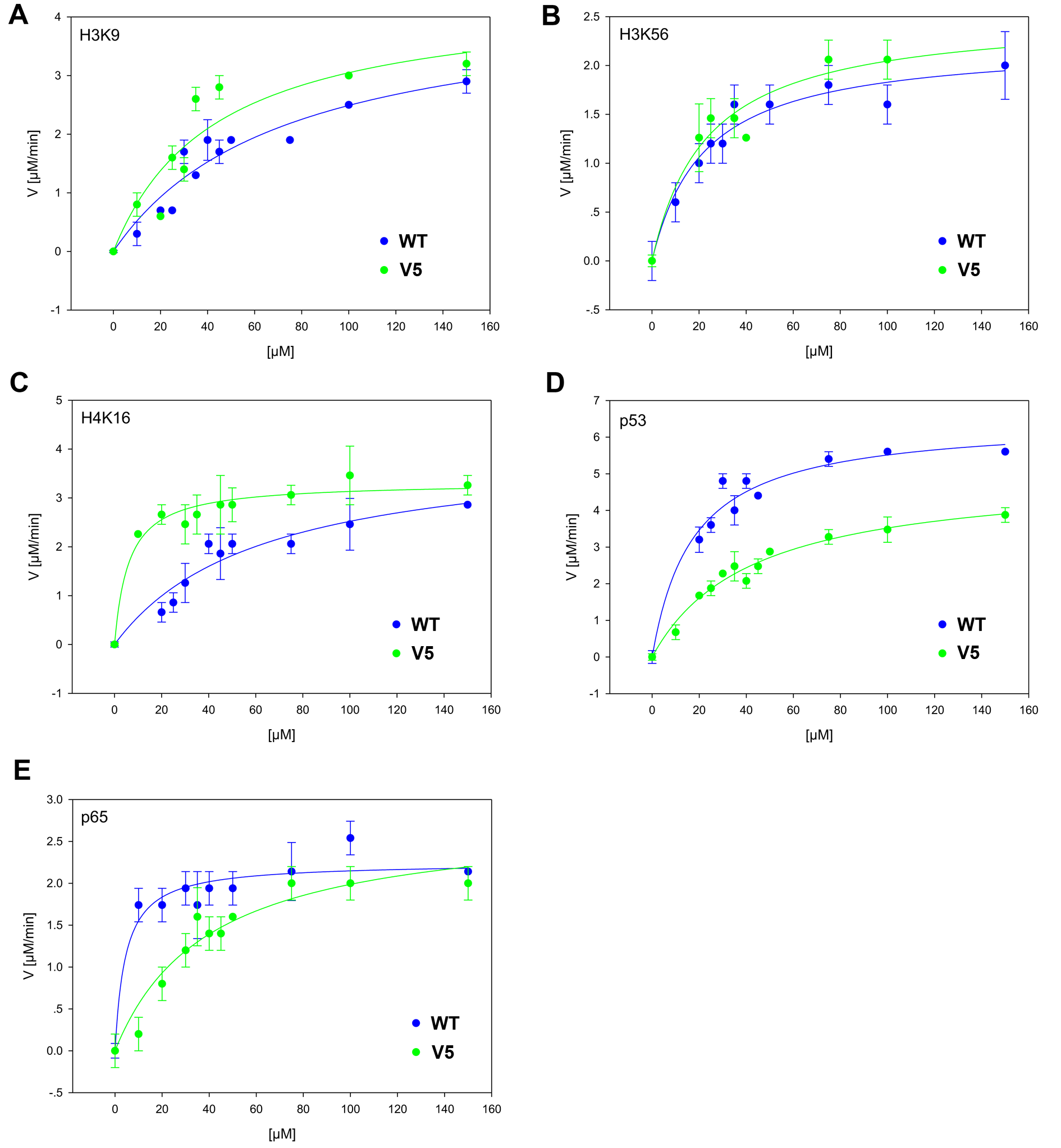
**

**Fig. S11: Michaelis Menten (MM) analysis of the WT and V5 hSIRT1 variants**. Initial rates were measured by the continuous ammonia assay following incubation of the hSIRT1 variants with different concentrations of acetylated peptides, (**A**) H3K9Ac, (**B**) H3K56Ac, (**C**) H4K16Ac, (**D**) p53 containing K382Ac and (**E**) RelA/p65 containing K310Ac. The initial rates were plotted against substrate concentration and fitted to the MM equation to derive the k_cat_ and K_M_ parameters reported in **Table 1**. Each experiment was performed in triplicate and representative experiment is shown. All peptide sequences are shown in **Table S4**.


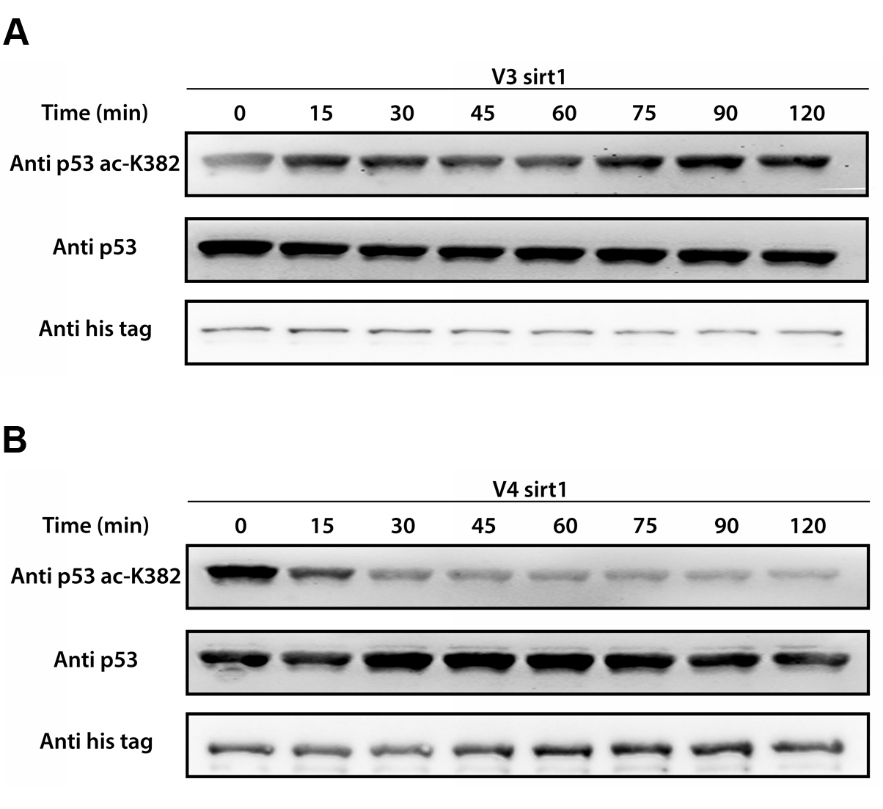


**Fig. S12: The activity of V3 and V4 with native acetylated p53.** Representative western blot blots for the analysis of V3 (**A**) and V4 (**B**) activity with native p53 K382Ac. Detection of p53-K382Ac, p53 and hSIRT1 levels was performed using anti-K382Ac antibody, anti-p53 antibody and anti-6xHis antibody, respectively. Western blot kinetic analysis of V3 and V4 activity toward p53-K382Ac in the context of native p53 are shown in **Fig. 5**.


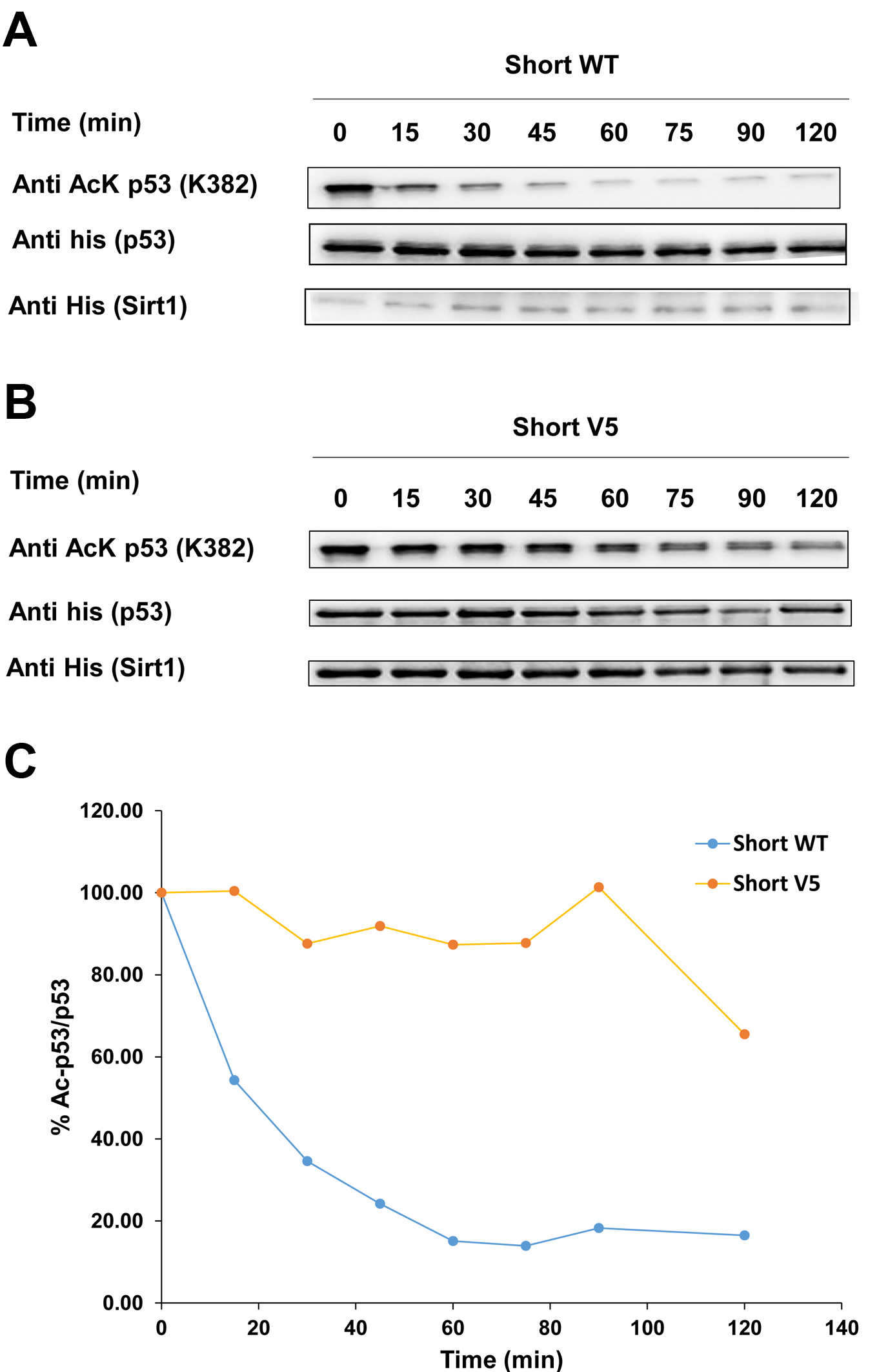


**Fig. S13: The activity of short truncated versions of WT and V5 with native acetylated p53.** Western blots for the analysis of short WT (**A**) and short V5 variant (**B**) activity with native p53 K382Ac. Detection of p53-K382Ac, p53 and hSIRT1 levels was performed using anti-p53 K382Ac antibody and anti-6xHis antibody for p53 and Sirt1. (**C**) Western blot kinetic analysis of short WT and short V5 variant activity toward p53-K382Ac in the context of native p53. The time dependent decrease in K382Ac signal was normalized to time 0 and shown as the percentage of p53-K382Ac/p53 bands. Band intensities were quantified by Image J.

**
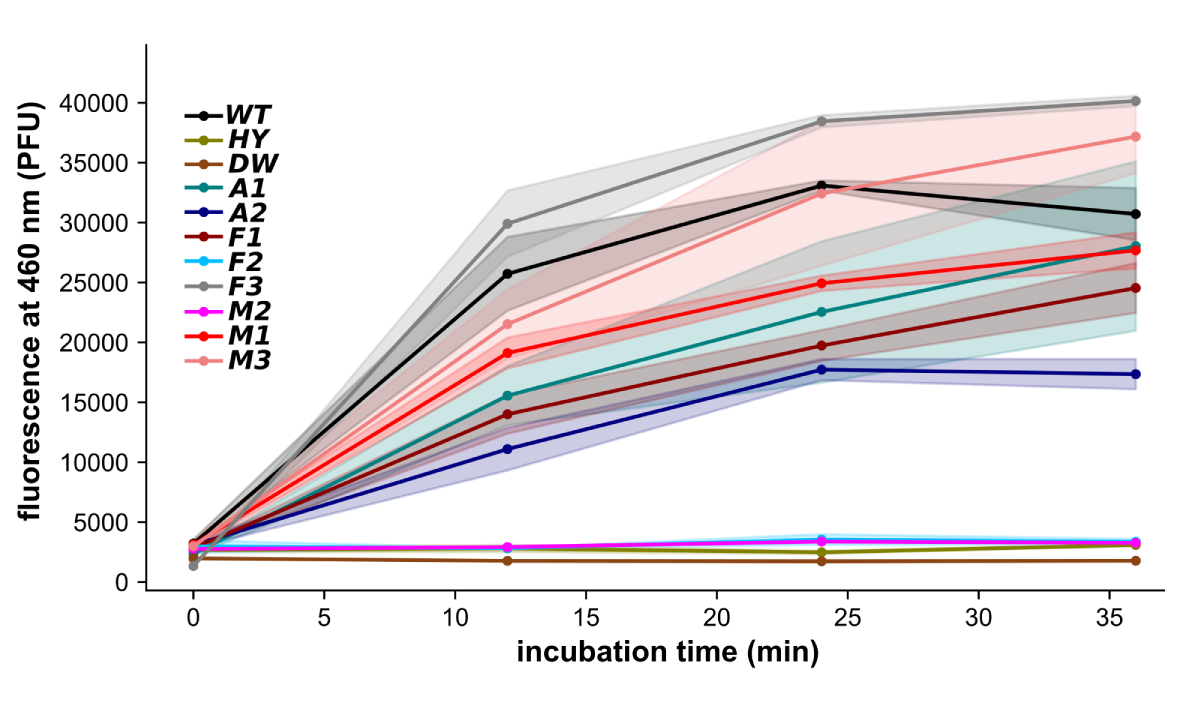
**

**Fig. S14: Fluor De lyse (FDL) analysis of hSIRT1 ASR mutants with protected acetyl-lysine-AMC substrate.**  Representative curves of fluorescent signal (PFU) at 460 nm for deacetylation of Boc-AcK-AMC. Shaded regions around curves show the standard error of the mean (SEM) for 2 technical replicates. WT served as the positive control; H363Y (HY; a mutation to the catalytically essential histidine at position 363) served as the the negative control, alongside DW (deionised water).





**Fig. S15: Representative western-blot analysis of SIRT1 ASR variants' activity with native H4K16Ac and p53 K382Ac.** Activity with histones (H4K16Ac) was measured up to 3 min (180 sec) of incubation while activity with p53 K382Ac was measured up to 120 min of incubation. All assays were performed as described in **Materials and Methods** and in experiments shown in **Fig. 6**.

**Table S1. Statistical comparison between constrained and unconstrained SIRT1 phylogenies.**

| Tree | logL | deltaL | bp-RELL | p-KH | p-SH | c-ELW | p-AU |
| --- | --- | --- | --- | --- | --- | --- | --- |
| ML tree | -24616.53285 | 0 | 0.661 | 0.655 | 1 | 0.66 | 0.654 |
| Constrained tree | -24626.61395 | 10.081 | 0.339 | 0.345 | 0.345 | 0.34 | 0.346 |

logL: log-likelihood; deltaL: logL difference from the tree with maximal logL in test (i.e. the ML tree); bp-RELL: bootstrap proportion using RELL method (Kishino et al. 1990), i.e. proportion of trees that returned this topology in the 10,000 RELL bootstrap pseudoreplicate trees used in the test; p-KH: p-value of one sided Kishino-Hasegawa test; p-SH:pvalue of Shimodaira-Hasegawa test; c-ELW: Expected LikelihoodWeight; p-AU: p-value of approximately unbiased (AU) test. All tests indicate that the constrained tree topology is not significantly different from the ML tree topology. Tests were performed using 10,000 resamplings (i.e. pseudoreplicate trees) with the RELL method.

**Table S2: Documented hSIRT1 substrates and acetylation sites (analyzed in Fig. 1).**

|  | **Substrate** | **Deacetylated lysine^a^** | **Reference (PMID)** |
| --- | --- | --- | --- |
| 1 | Histone H4 | K16 | 10693811 |
| 2 | Histone H3 | K9, K56 | 19879981, 19411844 |
| 3 | Tip60 | ND | 20100829 |
| 4 | PCAF | ND | 19188449 |
| 5 | ATG7 | ND | 25316028 |
| 6 | XPA/XPC | K67 | 20670893 |
| 7 | STK11(LKB11) | ND | 18687677 |
| 8 | MEF2 | K424 | 16166628 |
| 9 | Apex1 | K7 | 20699270 |
| 10 | Ku70 | K539/K542 | 15023334 |
| 11 | WRN | ND | 20428248 |
| 12 | MyoD | ND | 22771996 |
| 13 | p300/CBP | K1020 | 15632193 |
| 14 | HIF-1α | K647 | 20620956 |
| 15 | FOXO3 | ND | 21841822 |
| 16 | FOXO1 | ND | 15220471 |
| 17 | PPARγ | K268 | 22863012 |
| 18 | p53 | K382 | 11672523 |
| 19 | c-Myc | ND | 22190494 |
| 20 | RelA | K310 | 18419308 |
| 21 | IRS2 | ND | 18590691 |
| 22 | HIC1 | K314 | 17283066 |
| 23 | NBN(NBS1) | ND | 17612497 |
| 24 | CIITA | ND | 21890893 |
| 25 | PML | K487 | 22274616 |

^a^ND- non determined

**Table S3: Kinetic parameters of WT and V3-5 with different acetylated peptides^a^.**

**
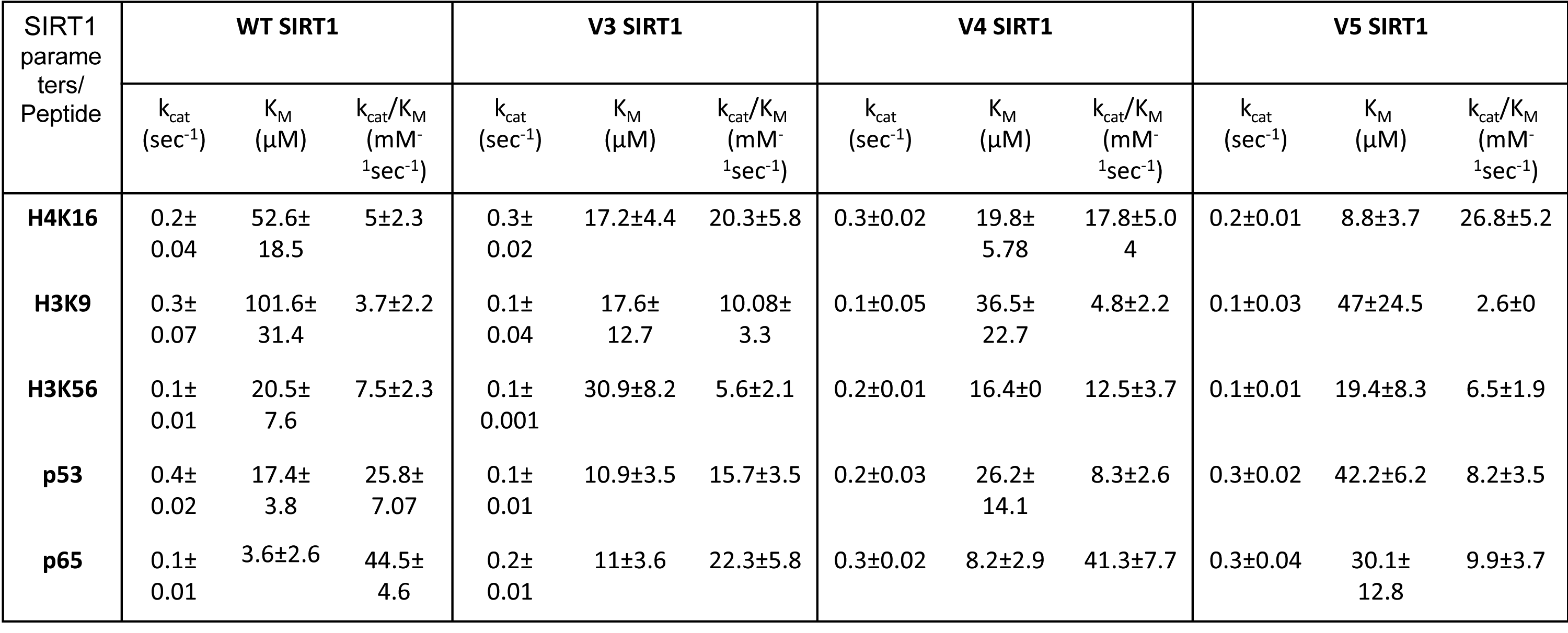
**

^a^Parameters were derived from fitting the kinetic measurements of the different hSIRT1 variants to the MM equation.

**Table S4: Peptide sequences of SIRT1 substrates used in this study**

| **SIRT1 substrates** | **AcLys in sequence** | **Peptide Sequence** |
| --- | --- | --- |
| H4 | K16 | KGGAK(Ac)RHRKVLRD |
| H3 | K9 | TKQTARK(Ac)STGGKAP |
| H3 | K56 | REIRRYQK(Ac)STELLIR |
| p53 | K382 | QSTSRHKK(Ac)LMFKTEG |
| RelA/p65 | K310 | RKRTYETFK(Ac)SIMKKSPF |

**References**

Akiva E, Brown S, Almonacid DE, Barber AE, Custer AF, Hicks MA, Huang CC, Lauck F, Mashiyama ST, Meng EC, et al. 2014. The Structure-Function Linkage Database. Nucleic Acids Res. 42:D521-30.

Akiva E, Copp JN, Tokuriki N, Babbitt PC. 2017. Evolutionary and molecular foundations of multiple contemporary functions of the nitroreductase superfamily. Proc. Natl. Acad. Sci. 114:E9549–E9558.

Altschul SF, Gish W, Miller W, Myers EW, Lipman DJ. 1990. Basic local alignment search tool. J. Mol. Biol. 215:403–410.

Arbely E, Natan E, Brandt T, Allen MD, Veprintsev DB, Robinson C V, Chin JW, Joerger AC, Fersht AR. 2011. Acetylation of lysine 120 of p53 endows DNA-binding specificity at effective physiological salt concentration. Proc. Natl. Acad. Sci. U. S. A. 108:8251–8256.

Atkinson HJ, Morris JH, Ferrin TE, Babbitt PC. 2009. Using Sequence Similarity Networks for Visualization of Relationships Across Diverse Protein Superfamilies.Jordan IK, editor. PLoS One 4:e4345.

Balakrishnan S, Kamisetty H, Carbonell JG, Lee S-I, Langmead CJ. 2011. Learning generative models for protein fold families. Proteins 79:1061–1078.

Barber AE, Babbitt PC. 2012. Pythoscape: a framework for generation of large protein similarity networks. Bioinformatics 28:2845–2846.

Bergsten J. 2005. A review of long-branch attraction. Cladistics 21:163–193.

Finn RD, Attwood TK, Babbitt PC, Bateman A, Bork P, Bridge AJ, Chang H-Y, Dosztányi Z, El-Gebali S, Fraser M, et al. 2017. InterPro in 2017-beyond protein family and domain annotations. Nucleic Acids Res. 45:D190–D199.

Garrity J, Gardner JG, Hawse W, Wolberger C, Escalante-Semerena JC. 2007. N-lysine propionylation controls the activity of propionyl-CoA synthetase. J. Biol. Chem. 282:30239–30245.

Gerlt JA, Bouvier JT, Davidson DB, Imker HJ, Sadkhin B, Slater DR, Whalen KL. 2015. Enzyme Function Initiative-Enzyme Similarity Tool (EFI-EST): A web tool for generating protein sequence similarity networks. Biochim. Biophys. Acta 1854:1019–1037.

Gertman O, Omer D, Hendler A, Stein D, Onn L, Khukhin Y, Portillo M, Zarivach R, Cohen HY, Toiber D, et al. 2018. Directed evolution of SIRT6 for improved deacylation and glucose homeostasis maintenance. Sci. Rep. 8.

Huerta-Cepas J, Szklarczyk D, Forslund K, Cook H, Heller D, Walter MC, Rattei T, Mende DR, Sunagawa S, Kuhn M, et al. 2016. eggNOG 4.5: a hierarchical orthology framework with improved functional annotations for eukaryotic, prokaryotic and viral sequences. Nucleic Acids Res. 44:D286-93.

Johnson LS, Eddy SR, Portugaly E. 2010. Hidden Markov model speed heuristic and iterative HMM search procedure. BMC Bioinformatics 11:431.

Kalyaanamoorthy S, Minh BQ, Wong TKF, von Haeseler A, Jermiin LS. 2017. ModelFinder: fast model selection for accurate phylogenetic estimates. Nat. Methods 14:587–589.

Katoh K. 2002. MAFFT: a novel method for rapid multiple sequence alignment based on fast Fourier transform. Nucleic Acids Res. 30:3059–3066.

Kishino H, Miyata T, Hasegawa M. 1990. Maximum likelihood inference of protein phylogeny and the origin of chloroplasts. J. Mol. Evol. 31:151–160.

Knyphausen P, de Boor S, Kuhlmann N, Scislowski L, Extra A, Baldus L, Schacherl M, Baumann U, Neundorf I, Lammers M. 2016. Insights into Lysine Deacetylation of Natively Folded Substrate Proteins by Sirtuins. J. Biol. Chem. 291:14677–14694.

Le SQ, Gascuel O. 2008. An improved general amino acid replacement matrix. Mol. Biol. Evol. 25:1307–1320.

Li W, Godzik A. 2006. Cd-hit: a fast program for clustering and comparing large sets of protein or nucleotide sequences. Bioinformatics 22:1658–1659.

Minh BQ, Nguyen MAT, von Haeseler A. 2013. Ultrafast approximation for phylogenetic bootstrap. Mol. Biol. Evol. 30:1188–1195.

NCBI Resource Coordinators R, Barrett T, Beck J, Benson DA, Bollin C, Bolton E, Bourexis D, Brister JR, Bryant SH, Canese K, et al. 2018. Database resources of the National Center for Biotechnology Information. Nucleic Acids Res. 46:D8–D13.

Nguyen L-T, Schmidt HA, von Haeseler A, Minh BQ. 2015. IQ-TREE: a fast and effective stochastic algorithm for estimating maximum-likelihood phylogenies. Mol. Biol. Evol. 32:268–274.

Pei J, Kim B-H, Grishin N V. 2008. PROMALS3D: a tool for multiple protein sequence and structure alignments. Nucleic Acids Res. 36:2295–2300.

Punta M, Coggill PC, Eberhardt RY, Mistry J, Tate J, Boursnell C, Pang N, Forslund K, Ceric G, Clements J, et al. 2012. The Pfam protein families database. Nucleic Acids Res. 40:D290-301.

dos Reis M, Thawornwattana Y, Angelis K, Telford MJ, Donoghue PCJ, Yang Z. 2015. Uncertainty in the Timing of Origin of Animals and the Limits of Precision in Molecular Timescales. Curr. Biol. 25:2939–2950.

Shannon P, Markiel A, Ozier O, Baliga NS, Wang JT, Ramage D, Amin N, Schwikowski B, Ideker T. 2003. Cytoscape: a software environment for integrated models of biomolecular interaction networks. Genome Res. 13:2498–2504.

Smith BC, Hallows WC, Denu JM. 2009. A continuous microplate assay for sirtuins and nicotinamide-producing enzymes. Anal. Biochem. 394:101–109.

Sonnhammer ELL, Östlund G. 2015. InParanoid 8: orthology analysis between 273 proteomes, mostly eukaryotic. Nucleic Acids Res. 43:D234-9.

Spatafora JW, Chang Y, Benny GL, Lazarus K, Smith ME, Berbee ML, Bonito G, Corradi N, Grigoriev I, Gryganskyi A, et al. 2016. A phylum-level phylogenetic classification of zygomycete fungi based on genome-scale data. Mycologia 108:1028–1046.

Stewart JJ, Lee CY, Ibrahim S, Watts P, Shlomchik M, Weigert M, Litwin S. 1997. A Shannon entropy analysis of immunoglobulin and T cell receptor. Mol. Immunol. 34:1067–1082.

UniProt Consortium. 2013. Update on activities at the Universal Protein Resource (UniProt) in 2013. Nucleic Acids Res. 41:D43-7.

Wang XW, Tan NS, Ho B, Ding JL. 2006. Evidence for the ancient origin of the NF- B/I B cascade: Its archaic role in pathogen infection and immunity. Proc. Natl. Acad. Sci. 103:4204–4209.

Yang Z. 2007. PAML 4: Phylogenetic analysis by maximum likelihood. Mol. Biol. Evol. 24:1586–1591.

Yang Z. 2014. Molecular Evolution. Oxford University Press

Yuan LW, Giordano A. 2002. Acetyltransferase machinery conserved in p300/CBP-family proteins. Oncogene 21:2253–2260.

Zerbino DR, Achuthan P, Akanni W, Amode MR, Barrell D, Bhai J, Billis K, Cummins C, Gall A, Girón CG, et al. 2018. Ensembl 2018. Nucleic Acids Res. 46:D754–D761.
